# Stage-specific remodeling of the pulmonary microenvironment during *Paragonimus proliferus* infection in a rat model

**DOI:** 10.1371/journal.pntd.0014760

**Published:** 2026-09-25

**Authors:** Danhong Cheng, Lei Zhang, Le Sun, Min Yin, Yu Wang, Shuwen Yang, Xing Yan, Xiaoyan Zhu, Cuiying Li, Weixun Chunyu

**Affiliations:** 1 Yunnan Provincial Key Laboratory of Public Health and Biosafety & Department of Pathogen Biology and Immunology, Faculty of Basic Medical Science, Kunming Medical University, Kunming, Yunnan, PR China; 2 School of Medicine, Yunnan University, Kunming, Yunnan, PR China; 3 Department of Anesthesiology, Kunming Medical University First Clinical College, First Affiliated Hospital of Kunming Medical University, Kunming, Yunnan, PR China; Uniformed Services University: Uniformed Services University of the Health Sciences, UNITED STATES OF AMERICA

## Abstract

Paragonimiasis, a food-borne zoonosis caused by *Paragonimus* spp., can cause severe pulmonary inflammation and fibrosis. However, relationships among the host, parasite and lung microbiome at defined infection stages remain poorly understood. We compared independent groups of rats sampled at 14, 28 and 42 days post-infection (dpi), together with a separate group sampled after triclabendazole (TCBZ) treatment. Histopathological and molecular analyses were combined with multi-region (5R) 16S rRNA gene sequencing and fluorescence *in situ* hybridization (FISH). Groups sampled at later post-infection time points showed greater pulmonary inflammation and collagen deposition, with the highest values in Pp-42d; both outcomes were lower in Pp-TCBZ than in Pp-42d. Th1-, eosinophil-, Th2- and Treg- associated markers differed among experimental groups. TLR4/NF-κB -related protein expression and pulmonary microbial profiles also differed among groups including enrichment of taxa such as *Lactobacillus* in infected animals. FISH detected bacterial signals within inflammatory lesions and *Lactobacillus*-associated signals spatially associated with parasite eggs. These repeated cross-sectional findings identify stage-associated differences in pulmonary pathology, immune markers and microbiota during *P. proliferus* infection, together with lower pathological measures in the Pp-TCBZ group than in the untreated Pp-42d group. The egg-associated bacterial signals warrant further investigation but do not establish a functional host-parasite-microbiome mechanism.

## Author summary

Paragonimiasis is a food-borne parasitic disease caused by lung flukes (*Paragonimus* species). It can resemble tuberculosis, which may delay appropriate treatment and contribute to lung damage. In this study, we compared separate groups of rats sampled at 14, 28 and 42 days after infection with *Paragonimus proliferus*, together with a separate group sampled after treatment with triclabendazole. Rats sampled at later post-infection time points had more extensive lung inflammation and scarring, with the greatest abnormalities in the 42-day group. The treated group had less pathological injury than the untreated 42-day group. Markers associated with several immune-response profiles, the TLR4/NF-κB pathway and the lung microbiome differed among the experimental groups. Fluorescence microscopy also detected *Lactobacillus* associated signals near parasite eggs. However, this spatial association does not establish a functional interaction. Overall, our findings provide a group-level description of pulmonary pathology, immune markers, and microbial profiles in independent rat groups sampled at selected infection stages and identify questions for future mechanistic investigation.

## Introduction

Paragonimiasis, a zoonotic parasitic disease, is caused by trematodes belonging to the genus *Paragonimus* [1]. This disease continues to pose a significant public health challenge in areas, such as Asia, Africa and the Americas, primarily because of the consumption of raw or insufficiently cooked freshwater crab harboring metacercariae [2]. Globally, paragonimiasis affects an estimated 23 million people, with distinct *Paragonimus* species distributed across continents, each capable of infecting humans [3]. Regionally, the disease burden varies considerably. In endemic areas of Africa, such as Cameroon and Nigeria, prevalence rates can exceed 10% in certain communities, highlighting significant local transmission [4]. In Asia, particularly in China, the Democratic People's Republic of Korea, the Republic of Korea, Japan, and Vietnam, paragonimiasis remains a persistent health concern, with sporadic outbreaks often linked to the consumption of regional culinary practices involving raw or pickled crab [1]. In the Americas, despite ongoing transmission in countries like Ecuador, Peru, and Mexico, the disease is frequently under-recognized, contributing to substantial diagnostic delays [5]. The pulmonary manifestation of paragonimiasis is clinically characterized by chronic inflammation, granuloma formation, and progressive pulmonary fibrosis, which frequently mimics the clinical and radiological presentation of pulmonary tuberculosis [6,7]. Consequently, misdiagnosis is prevalent, often resulting in delayed therapeutic interventions and serious complications, such as progressive lung damage [1,8]. Although praziquantel remains the first-line drug for treating trematode infections [9], a retrospective clinical analysis of pediatric paragonimiasis showed that triclabendazole demonstrates superior efficacy against refractory manifestations [10].

*Paragonimus* species follow a complex life cycle involving freshwater gastropods and crab as intermediate hosts [1]. Humans and other mammals are infected by consuming infected freshwater crab harboring metacercariae that excyst in the intestine and migrate to the lungs, where they mature into adult worms [2]. The eggs produced by the adult worms are expelled through sputum or feces, completing the life cycle [11]. Several critical time points occur during *P. proliferus* infection. At 14 days post-infection (14 dpi), larvae migrate to the pulmonary tissue and establish residence within the host lungs. By 28 dpi, the parasites develop and mature. At 42 dpi, oviposition begins and a number of eggs are released. The definitive hosts of most *Paragonimus* species are humans or carnivorous mammals, making it difficult and costly to establish experimental infections in laboratory animals. *P. proliferus*, which is primarily endemic in Yunnan Province of China and parts of Vietnam, can successfully infect Sprague-Dawley (SD) rats in the laboratory, providing a useful model for studying the pathogenesis of paragonimiasis, even though its definitive hosts in the wild remain unconfirmed [12,13]. From an epidemiological perspective, the identification of definitive hosts is critical, as they serve as key reservoirs that sustain the parasite's life cycle in nature and contribute to the contamination of freshwater bodies with eggs, thereby driving transmission to intermediate hosts and, ultimately, to humans [3,4].

In general, parasitic helminth infections are associated with Th2 responses and regulatory T-cell (Treg) activation, together with modulation of Th1-mediated immunity [14]. These immune profiles may influence pulmonary inflammation and parasite persistence, but their roles can vary by parasite species, tissue and infection context. Although the life cycle of *P. proliferus* is well documented, it remains unclear whether pulmonary pathology and host immune markers differ among animals sampled at defined stages of infection.

The lung microbiota has been identified as a crucial element in shaping immune responses and tissue remodeling during the course of infections [15,16]. Our earlier research revealed alterations in the pulmonary microbiota linked to *P. proliferus* infection [16]. Nevertheless, the impact of *P. proliferus* infection on the lung microbiome and its interplay with host immune responses is still largely uninvestigated.

Consequently, the present study compared pulmonary inflammation and fibrosis, TLR4/NF-κB -related proteins and lung microbial profiles among independent rat groups sampled at 14, 28 and 42 dpi and a separate TCBZ-treated group. Because the design was repeated cross-sectional, the findings were interpreted as group-level, stage-associated differences rather than within-animal temporal trajectories. By integrating histopathological analysis, immune profiling and multi-region microbiome sequencing, we aimed to characterize pulmonary changes associated with defined stages of *P. proliferus* infection and those observed in the Pp-TCBZ group. Pulmonary pathological injury and fibrosis were designated as the primary outcome domains and were assessed using the relative ATS lung injury score and collagen area fraction (CAF), respectively. The primary null hypotheses were that (i) relative ATS lung injury scores and (ii) CAF values would not differ among the Ctrl, Pp-14d, Pp-28d, Pp-42d and Pp-TCBZ groups. qRT-PCR, ELISA and TLR4/NF-κB pathway outcomes were classified as secondary outcomes, whereas pulmonary microbiome outcomes were classified as exploratory (Table D in S1 Appendix).

## Materials and methods

### Ethics statement

All animal experiments were conducted in accordance with institutional and national guidelines for the care and use of laboratory animals and were approved by the Animal Ethics Committee of Kunming Medical University (Approval No. KMMUX2025070214).

### Collection of *P. proliferus* metacercariae

Freshwater crabs (*Potamon chinghungense* Dai, Song, He, Cao, Xu & Zhong, 1975 [17]) were collected from Mengla County (Xishuangbanna Dai Autonomous Prefecture, Yunnan Province, China), a known endemic region for *P. proliferus.* Under a stereomicroscope, the metacercariae were examined, manually separated from the freshwater crab tissues and then transferred into sterile saline for later use. To ensure parasite viability, the collected metacercariae were maintained in saline and used to infect SD rats within a 24-hour period. Representative specimens were photographed and documented using a light microscope equipped with a digital imaging system (E3ISPM06300KPB, E3ISPM, China). The metacercariae were identified as *P. proliferus* Hsia & Chen, 1964, based on previously described morphological criteria [18].

### Animals and experimental infection

Male SD rats (8–12 weeks old, 275 ± 25 g) were purchased from the Experimental Animal Center of Kunming Medical University. The use of male SD rats was based on previous experimental models of *Paragonimus* infection and their ability to establish reproducible laboratory infections with *P. proliferus*, making them suitable for investigating host–parasite interactions under controlled experimental conditions. Animals were maintained under specific pathogen-free (SPF) conditions in individually ventilated cages (IVCs) with controlled temperature (24 ± 2°C), relative humidity (50–60%) and a 12 h light/dark cycle. Rats were group-housed with six animals per cage, with one cage assigned to each experimental group, under identical environmental conditions. Bedding, chow, and water were replaced according to the same schedule for all groups. Standard laboratory chow and water were provided *ad libitum*. Rats were acclimatized for 14 days before experimentation.

For experimental infection, each rat was orally gavaged with 10 viable metacercariae suspended in 1 mL sterile physiological saline. Control animals received an equal volume of sterile physiological saline by oral gavage.

All animal procedures were conducted in accordance with institutional and national guidelines for laboratory animal care and use and were approved by the Animal Ethics Committee of Kunming Medical University (Approval No. KMMUX2025070214).

### Experimental design and antiparasitic treatment

The overall experimental design and workflow were schematically illustrated in Fig 1A. The repeated cross-sectional design included five sampled groups: an uninfected control group (Ctrl), infected groups sampled at 14, 28 and 42 days post-infection (Pp-14d, Pp-28d and Pp-42d), and a triclabendazole-treated group (Pp-TCBZ). Six animals per group were used for pulmonary microbiome profiling, five animals per group were analyzed for histopathology, qRT-PCR, and ELISA, and three independent lung tissue samples per group were analyzed by Western blotting. Before infection, rats were randomly assigned to experimental groups and housed in identical cages, with six animals per cage. Animals within the same experimental group were maintained together throughout the experiment. All groups were kept under the same environmental conditions, including identical cage specifications, bedding, chow, and water replacement schedules, to minimize potential cage-related environmental variation. At each predefined sampling time point, animals from the corresponding group were euthanized for sample collection. Triclabendazole treatment was initiated at 42 dpi (10 mg/kg/day by oral gavage) and continued for 5 consecutive days; the Pp-TCBZ group was sampled at 60 dpi after a post-treatment recovery interval.

**Fig 1:**
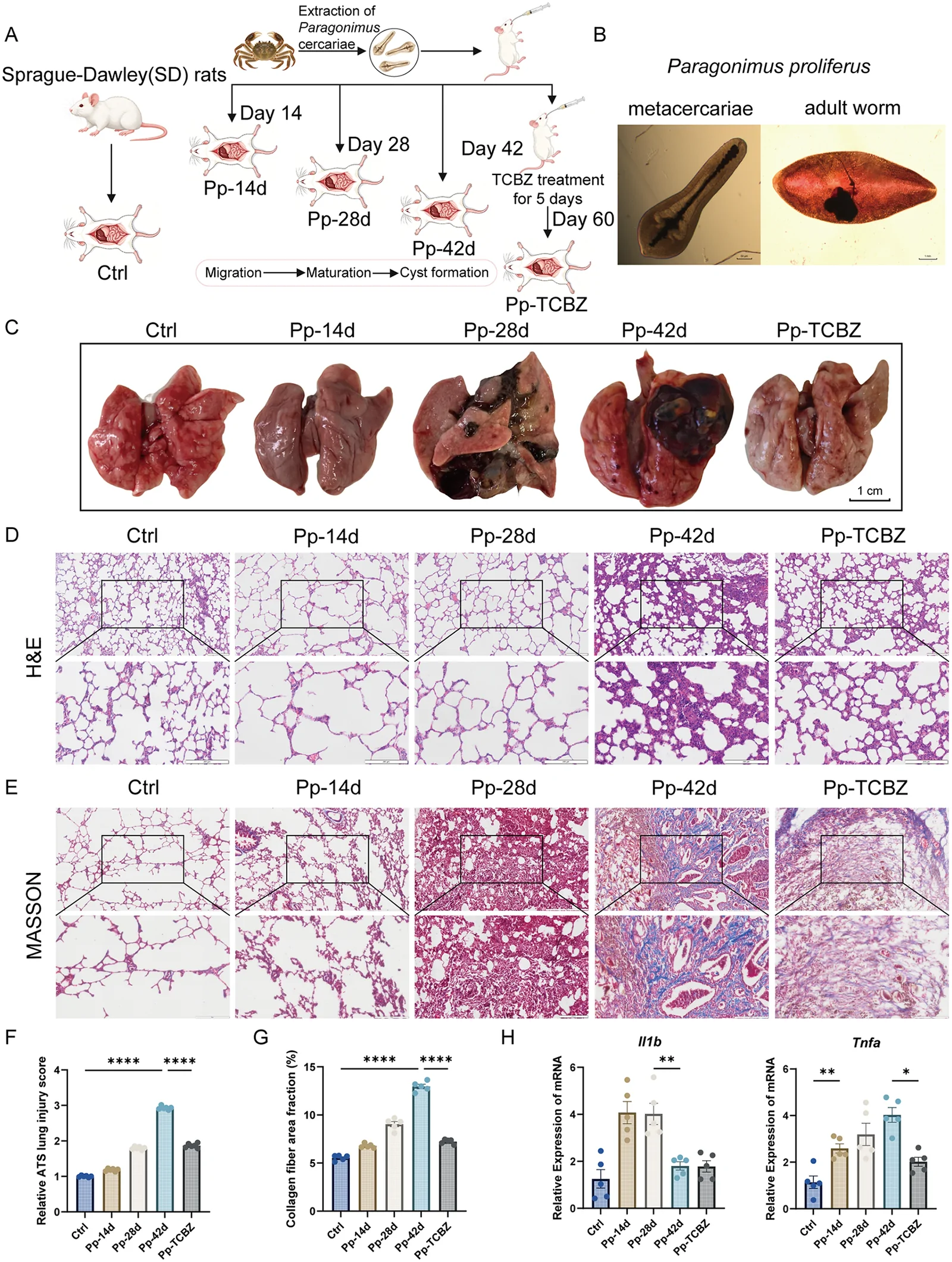
Pulmonary pathological features across *P. proliferus* infection stages and after triclabendazole treatment. (A) Schematic diagram of the experimental design. Sprague-Dawley rats were infected with *P. proliferus* metacercariae and sacrificed at different time points (Pp-14d, Pp-28d and Pp-42d). In the treatment cohort, triclabendazole (TCBZ) administration was initiated at 42 dpi and continued for 5 consecutive days, followed by a 12-day recovery period before sampling at 60 dpi (Pp-TCBZ). (B) Representative images of *P. proliferus* metacercariae isolated from freshwater crabs and adult worms collected from infected rats. (C) Representative photographs of lung tissues showing gross morphological differences among groups sampled at 14, 28 and 42 dpi and after TCBZ treatment. (D) Representative hematoxylin and eosin (H&E) staining showing greater inflammatory cell infiltration, alveolar septal thickening and structural disruption in groups sampled at later post-infection time points. The TCBZ-treated group showed less extensive pathological damage than untreated infected rats at 42 dpi. (E) Masson's trichrome staining showing differences in collagen deposition among groups sampled at 14, 28 and 42 dpi. Collagen deposition was less extensive in the TCBZ-treated group than in untreated infected rats at 42 dpi. (F) Relative ATS lung injury scores. ATS lung injury scores were averaged across three fields and two blinded observers for each animal and then normalized to the mean of the Ctrl group. (G) Quantification of collagen fiber area fraction based on Masson staining. (H) Relative mRNA expression levels of *Il1b* and *Tnfa* in lung tissues determined by qRT-PCR. Data are presented as mean ± SEM (*n* = 5 per group for pathological and molecular analyses) unless otherwise indicated. Each dot represents one individual animal. *P < 0.05, **P < 0.01, ***P < 0.001, ****P < 0.0001. Scale bars: H&E staining, 200 μm (including magnified regions); Masson's trichrome staining, 500 μm (overview) and 200 μm (magnified regions). The element of A was Created in BioRender. Cheng, D. (2026) *https://BioRender.com/2qshjav*.

### Sample collection, tissue processing and preservation

At the designated time points, rats were anesthetized by intraperitoneal injection of tribromoethanol (10 mL/kg, i.p., Nanjing Aibei Biotechnology Co., Ltd., Nanjing, China). Subsequently, the animals were euthanized by cervical dislocation. The thoracic cavity was opened and lung tissues were excised and rinsed with sterile saline. Lung tissue surrounding, but excluding, the parasitic cysts was carefully dissected under sterile conditions.

Portions of lung tissues were allocated for different analyses. Samples for histological examination were fixed in 4% paraformaldehyde for 24 h. Tissues for RNA and protein analyses were immediately snap-frozen in liquid nitrogen and stored at -80°C. A subset of the dissected lung tissue surrounding the parasitic cysts was used for microbial community analysis. Because lung tissue microbiome analysis involves low-biomass samples, contamination controls were incorporated throughout sample processing, DNA extraction, and PCR amplification. Sample processing was performed under aseptic conditions in a biosafety cabinet. Three negative controls were processed in parallel with the biological samples and submitted to the same sequencing workflow; environmental samples were also submitted in parallel for background monitoring. None of the three negative controls yielded a detectable PCR amplification product; consequently, no sequence reads or microbial taxa were recovered from these controls. Because no microbial features were detected in the negative controls, no features were removed from the biological dataset on the basis of negative-control screening, and no model-based decontamination algorithm was applied. Isolated parasites were collected, washed with PBS, and stored at -80°C for further analysis.

### Histological staining and analysis

For hematoxylin and eosin (H&E) staining, rehydrated sections were stained with hematoxylin to visualize nuclei, differentiated in acid alcohol, blued in tap water and counterstained with eosin to label cytoplasm and extracellular components. After dehydration through graded ethanol and clearing in xylene, sections were mounted with neutral resin. Images were acquired using a digital pathology slide scanning system (OLYMPUS SLIDEVIEW VS200). Alveolar structure, inflammatory cell infiltration and histopathological changes were quantified according to the ATS lung injury scoring criteria (Table A in S1 Appendix). Representative H&E-stained fields illustrating the item-level score categories are provided in Fig A in S1 Appendix. Three histological fields per animal were independently scored by two observers blinded to group allocation. For each observer, the field-level scores were averaged, and the mean score from the two observers was used as the animal-level score. Animal-level scores were normalized to the mean of the Ctrl group for statistical analysis and visualization. Inter-rater reliability was assessed using a two-way random-effects, absolute-agreement, average-measures intraclass correlation coefficient (ICC(2,2) = 0.9999).

For Masson’s trichrome staining, a commercial kit (Solarbio, China, Cat. No. G1340) was used according to the manufacturer’s protocol. Collagen fibers were stained blue, muscle fibers and cytoplasm red and nuclei black. The collagen area fraction was quantified from at least five random fields per section using ImageJ (NIH, USA).

### RNA Extraction and Quantitative Real-Time PCR (qRT-PCR)

Following the manufacturer's guidelines, total RNA was isolated from lung tissues employing the RNAiso Plus reagent (Takara, Japan). Subsequently, a NanoDrop spectrophotometer (Thermo Fisher Scientific, USA) was utilized to ascertain the concentration and purity of the extracted RNA.

Complementary DNA (cDNA) was synthesized using a reverse transcription kit (Thermo Fisher Scientific, USA). Quantitative real-time PCR was performed using PerfectStart Green qPCR SuperMix (TransGen Biotech, China, Cat. No. AQ601–02-V2) on a real-time PCR system (Applied Biosystems, USA). Primer sequences used for quantitative real-time PCR are listed in Table B in S1 Appendix.

Relative gene expression levels were normalized to *Gapdh* and calculated using the 2^ − ΔΔCt method.

### Enzyme-Linked Immunosorbent Assay (ELISA)

Lung tissues were homogenized in ice-cold PBS containing protease inhibitors and centrifuged at 12,000 × g for 15 min at 4°C to remove insoluble components. The supernatants were collected and used for subsequent ELISA analysis. Total protein concentrations in the lung tissue homogenates were determined using a Pierce BCA Protein Assay Kit (Thermo Scientific, USA, Cat. No. 23227) according to the manufacturer’s instructions. The protein concentrations of all samples were adjusted to 10 mg/mL with PBS prior to ELISA analysis.

The levels of interferon-gamma (IFN-γ), proteoglycan 2 (PRG2), interleukin-4 (IL-4), and interleukin-10 (IL-10) in lung tissue homogenates were quantified using commercial ELISA kits (JingMei, China) according to the manufacturer’s instructions. Briefly, samples and standards were added to antibody-precoated microplates and incubated, followed by incubation with enzyme-conjugated detection antibodies. After washing, stop solution was added for color development, and the reaction was terminated with a stop solution. Absorbance was measured at 450 nm using a microplate reader, and analyte concentrations were calculated from the corresponding standard curves. Five independent biological samples were analyzed per group, and each sample was assayed in duplicate.

### Protein extraction and western blot (WB) analysis

Lung tissues were homogenized using RIPA lysis buffer (Solarbio, Beijing, China; Cat. No. R0010) supplemented with phenylmethylsulfonyl fluoride (PMSF) and phosphatase inhibitor cocktail A (Beyotime, China; Cat. No. P1082). Tissue lysates were clarified by centrifugation at 12,000 × *g* for 15 min at 4°C, and the supernatants were collected for subsequent analysis. Protein concentrations were determined using a Pierce BCA Protein Assay Kit (Thermo Scientific, USA, Cat. No. 23227). For immunoblotting, equal amounts of protein (40 μg per lane) were loaded onto SDS-polyacrylamide gels. Proteins were separated by SDS-PAGE and transferred onto polyvinylidene difluoride (PVDF) membranes. Membranes were incubated overnight at 4°C with primary antibodies against TLR4 (Affinity Biosciences, Cat. No. AF7017), NF-κB p65 (Invitrogen, Cat. No. PA5–16545), and phosphorylated NF-κB p65 (Ser276, Invitrogen, Cat. No. PA5–37718). Additional primary antibodies targeted phosphorylated IκBα (Tyr305; Invitrogen, Cat. No. PA5–104851) and β-actin (Huabio, China, Cat. No. R1207-1). All five primary antibodies were reported by their manufacturers to be reactive with rat samples. Bands were detected at approximately 100 kDa for TLR4, 65 kDa for NF-κB p65 and phosphorylated NF-κB p65, 36 kDa for phosphorylated IκBα, and 42 kDa for β-actin. After incubation with appropriate HRP-conjugated secondary antibodies, the bands were visualized using a Bio-Rad imaging system and quantified using ImageJ software.

### Immunohistochemical (IHC) analysis

For immunohistochemical staining, paraffin sections were deparaffinized and rehydrated, followed by heat-induced antigen retrieval in citrate buffer (pH 6.0). Endogenous peroxidase activity was blocked using 3% hydrogen peroxide and nonspecific binding was blocked with normal serum.

Sections were incubated overnight at 4°C with primary antibodies against TLR4 (Affinity Biosciences, Cat. No. AF7017), NF-κB p65 (Invitrogen, Cat. No. PA5–16545), phosphorylated NF-κB p65 (Ser276) (Invitrogen, Cat. No. PA5–37718) and phosphorylated IκBα (Tyr305) (Invitrogen, Cat. No. PA5–104851). After washing, sections were incubated with HRP-conjugated secondary antibodies (MXB Biotechnologies, KIT-5020).

Immunoreactivity was visualized using 3,3′-diaminobenzidine (DAB) as the chromogen, followed by counterstaining with hematoxylin. Afterward, the slides were dehydrated, mounted and scanned using an Olympus digital slide scanning system. Representative images were then acquired and analyzed using ImageJ software.

### Fluorescence In Situ Hybridization (FISH)

FISH was applied to paraffin-embedded lung tissue sections to visualize pulmonary bacterial localization. Lung tissues fixed in 4% paraformaldehyde were embedded in paraffin and sectioned at a thickness of 4 μm. After deparaffinization in xylene and rehydration through a graded ethanol series, sections were rinsed in phosphate-buffered saline (PBS) to restore tissue hydration.

Hybridization was performed using the universal bacterial 16S rRNA probe EUB338 (5′-GCTGCCTCCCGTAGGAGT-3′), labeled with Cy3 at both the 5′ and 3′ ends. EUB338 was used at a final concentration of 10 ng/μL (Fubo Biotech, China; Cat. No. FBFP1360). A *Lactobacillus*-specific probe (5′-GCTACTCAACAAAGACGG-3′), labeled with Cy5 at both ends, was used at a final concentration of 20 ng/μL (Fubo Biotech; Cat. No. FBFP1353). Probe-containing hybridization buffer was applied to the sections, followed by incubation at 46°C for 4 h in a humidified, light-protected chamber.

Following hybridization, sections were washed twice for 15 min each at 46°C in prewarmed wash buffer. The buffer contained 0.9 M NaCl, 20 mM Tris-HCl (pH 7.4), 35% (v/v) formamide and 0.01% SDS. Sections were then washed twice with PBS for 5 min each and mounted with antifade mounting medium containing DAPI for nuclear counterstaining. Fluorescent signals were acquired using a laser-scanning confocal microscope with the instrument’s standard preset channel settings. DAPI was excited using the 405-nm laser and detected at 410–488 nm. Cy3-labeled EUB338 was excited using the 561-nm laser and detected at 570–620 nm. The Cy5-labeled *Lactobacillus*-specific probe was excited using the 640-nm laser and detected at 650–700 nm. For visualization, signals from the Cy5 channel were pseudocolored green in the merged images. Z-stack images were acquired to assess the spatial distribution of bacterial signals within lung tissues.

### 5R 16S rRNA gene sequencing and lung microbiome analysis

Total microbial DNA was extracted from lung tissues using the FastPure Stool DNA Isolation Kit (MJYH, Shanghai, China). Five hypervariable regions (V2, V3, V5, V6 and V8) were amplified in a multiplex PCR using region-specific primers (Table C in S1 Appendix) and Fast Pfu DNA polymerase. Amplicons from triplicate reactions per sample were pooled, purified, quantified and sequenced on an Illumina NextSeq 2000 platform by Majorbio Bio-Pharm Technology Co., Ltd. (Shanghai, China).

Raw reads were quality-filtered using fastp (v0.19.6) and aligned according to the corresponding primer sequences. Sequence denoising and ASV inference were performed using the DADA2 plugin in QIIME 2 (release 2024). SMURF was applied using its default settings to integrate region-specific read counts across the V2, V3, V5, V6 and V8 regions into unified microbial community profiles [19]. Taxonomic assignment was performed using the classify-sklearn Naive Bayes classifier against the SILVA 138.2 16S bacterial database, with a confidence threshold of 0.7 [20].

Bioinformatic analyses were conducted on the Majorbio Cloud platform. Across the 30 samples included in downstream analyses (*n* = 6 per group), denoising yielded a mean of 183,396 sequences per sample across the five amplified regions (range, 124,225–272,509; Table H in S1 Appendix). For downstream diversity analyses, all samples were rarefied to 124,225 sequences per sample, corresponding to the minimum denoised sequence count among the 30 analyzed samples. Rarefaction curves for the Sobs and Shannon indices are provided in Fig B in S1 Appendix. All 30 prespecified samples were retained, and none was excluded because of insufficient sequencing depth. The Sobs, Chao1, Shannon, and Simpson indices were calculated for alpha-diversity analysis. Beta diversity was assessed using species-level principal coordinate analysis (PCoA) based on Bray–Curtis dissimilarities. Differentially enriched taxa were identified using LEfSe with an LDA score threshold >3.5 and nominal *P* < 0.05.

### Statistical Analysis

Statistical analyses were performed using GraphPad Prism 10.4.1 and R software. Each animal was considered an independent biological replicate. Data are presented as mean ± SEM for normally distributed variables and as median with interquartile range for non-normally distributed variables. Normality and homogeneity of variance were assessed using the Shapiro–Wilk and Levene’s tests, respectively. Group comparisons were performed using ordinary one-way ANOVA, Welch’s ANOVA, or the Kruskal–Wallis test, as appropriate, followed by the corresponding multiple-comparisons procedure. For qRT-PCR data, statistical inference was based on ΔCt values, whereas 2^ − ΔΔCt values are presented for visualization. The Benjamini–Hochberg procedure was used to control the false discovery rate within prespecified outcome families (Table D in S1 Appendix). Pairwise comparisons were conducted only after a significant omnibus test. All tests were two-sided, and *P* < 0.05 was considered statistically significant after adjustment where applicable. Detailed test selection, multiple-comparison procedures, effect sizes, confidence intervals, post hoc power analysis for the primary outcomes, and raw and adjusted *P* values are provided in Tables E–G in S1 Appendix. Individual data points are shown on all bar graphs.

## Results

### 1. Pulmonary pathological changes following P. proliferus infection and triclabendazole treatment

Metacercariae were isolated from freshwater crabs, and adult worms were obtained from the lungs of rats at 42 days post-infection (Fig 1B). Gross examination revealed distinct pulmonary changes across the sampled post-infection time points (Fig 1C). At 14 dpi, the lung appeared grossly unremarkable, with no obvious pathological changes. At 28 dpi, the lungs were enlarged and displayed brown-black lesion areas. At 42 dpi, cysts containing adult worms were visible, and lung enlargement persisted. In the Pp-TCBZ group, the overall appearance of the lungs was largely restored, and no worms were detected.

H&E staining revealed stage-specific alterations in lung architecture across infection groups (Fig 1D). The Ctrl group exhibited intact alveolar structures with thin and uniform septa, whereas the Pp-14d group showed mild septal thickening. More pronounced structural alterations were observed in the Pp-28d group, with increased septal thickening and partial disruption of alveolar integrity. The most severe lesions were observed at the Pp-42d group, characterized by extensive inflammatory cell infiltration, marked destruction of alveolar architecture and focal consolidation. In the Pp-TCBZ group, inflammatory infiltration and tissue damage were markedly alleviated compared with Pp-42d group, although full restoration to control morphology was not achieved. Histopathological scores were higher in the Pp-28d and Pp-42d groups compared with the Ctrl and Pp-14d groups, with the highest scores observed in the Pp-42d group. The score was significantly lower in the Pp-TCBZ group than in the untreated Pp-42d group (Fig 1F).

Masson’s trichrome staining revealed stage-associated differences in collagen fiber deposition (Fig 1E). In Ctrl group, collagen fibers were largely confined to perivascular and peribronchial regions. Collagen deposition was mildly increased in the Pp-14d group, became more prominent and showed a patchy distribution in the Pp-28d group, and was greatest in the Pp-42d group, in which extensive dense collagen fibers were accompanied by marked distortion and loss of normal alveolar architecture. In contrast, the Pp-TCBZ group showed less collagen fiber deposition and better-preserved lung architecture than the Pp-42d group. Consistent with these observations, the collagen area fraction (CAF) was higher in the infected groups than in the Ctrl group and was highest in the Pp-42d group, whereas the CAF was significantly lower in the Pp-TCBZ group than in the Pp-42d group (Fig 1G).

To further characterize inflammatory activation at the transcriptional level, the relative mRNA expression levels of *Il1b* and *Tnfa* were measured in lung tissues (Fig 1H). *Il1b* expression was significantly lower in the Pp-42d group than in the Pp-14d and Pp-28d groups (both *P* < 0.01). *Il1b* expression in the Pp-TCBZ group was comparable to that in the Pp-42d group. The observed *Tnfa* expression levels increased in the order Pp-14d, Pp-28d, and Pp-42d, with the highest level observed in the Pp-42d group. *Tnfa* expression was significantly lower in the Pp-TCBZ group than in the Pp-42d group (*P* < 0.05). Collectively, these findings confirmed pulmonary infection by *P. proliferus* and the accompanying inflammatory responses.

The data showed differences in pulmonary inflammation and collagen deposition across the sampled post-infection time points. The most severe tissue damage and collagen deposition were observed at 42 dpi.

### 2. Stage-specific pulmonary immune responses during *P. proliferus* infection and after triclabendazole treatment

We measured the expression of Th1-, eosinophil-, Th2- and Treg-associated markers and cytokines at both the transcript and protein levels using qRT-PCR and ELISA. Infected lungs exhibited molecular changes consistent with anti-parasitic immune activation. At the transcriptional level, the Th1-associated genes *Tbx21* and *Ifng* were expressed at higher levels in Pp-14d than in Ctrl (*Tbx21*, *P* < 0.001; *Ifng*, *P* < 0.01; Fig 2A). *Tbx21* expression was lower in Pp-TCBZ than in Pp-42d (*P* < 0.05). In parallel, the eosinophil granule-associated genes *Epx* and *Prg2* were expressed at higher levels in Pp-14d than in Ctrl (*Epx*, *P* < 0.01; *Prg2*, *P* < 0.001; Fig 2B), consistent with enhanced eosinophil-linked inflammatory effector activity in the lung. *Prg2* expression was lower in Pp-TCBZ than in Pp-42d (*P* < 0.001). At the protein level, IFN-γ concentrations were higher in Pp-14d than in Ctrl (*P* < 0.001) and lower in Pp-TCBZ than in Pp-42d (*P* < 0.05). PRG2 concentrations were higher in Pp-28d than in Ctrl (*P* < 0.001) and lower in Pp-TCBZ than in Pp-28d (*P* < 0.05; Fig 2C).

**Fig 2:**
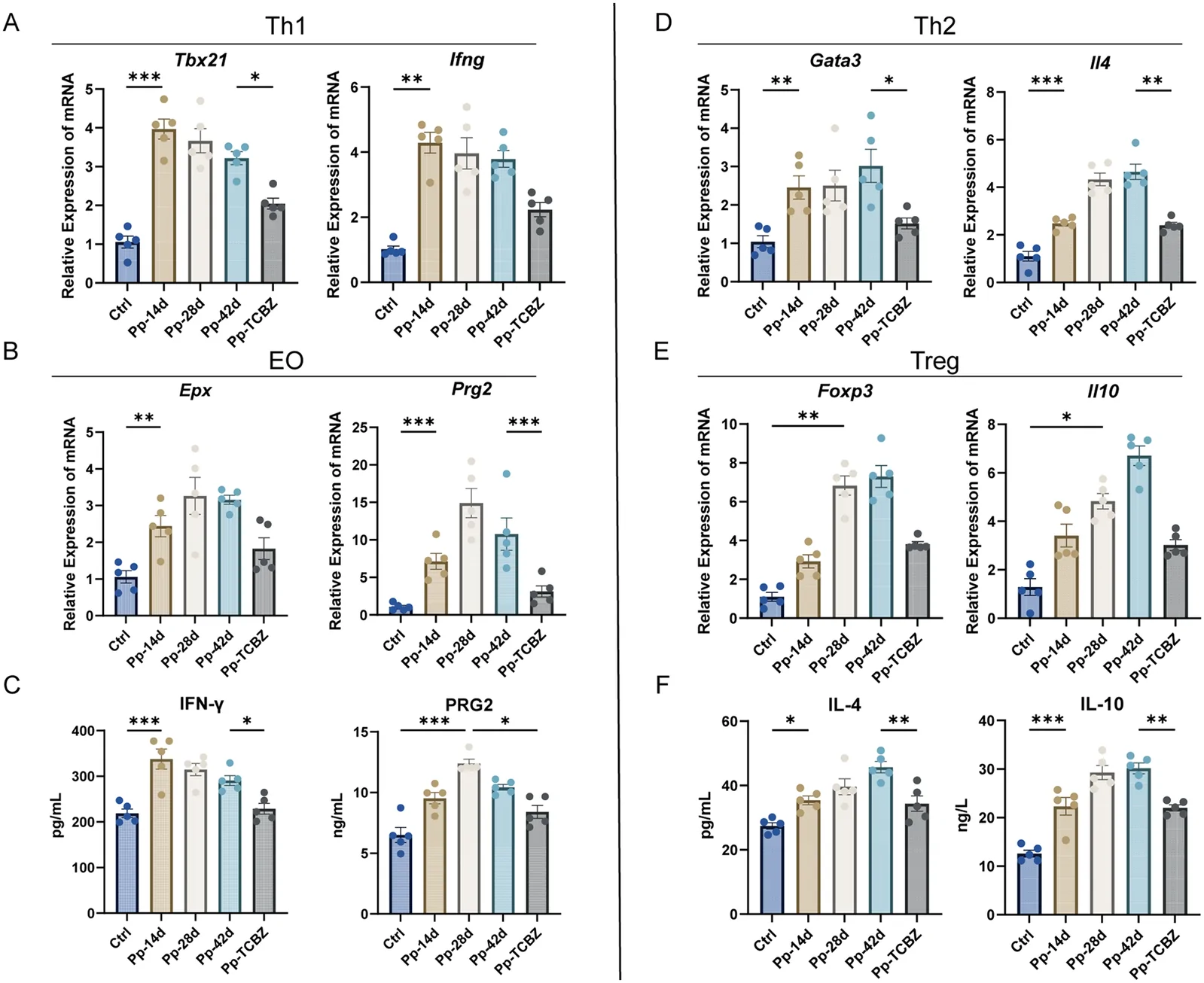
Pulmonary Th1/eosinophil, Th2 and Treg-associated immune profiles across *P. proliferus* infection stages and after triclabendazole treatment. (A–B) Relative mRNA expression levels of Th1-related genes (*Tbx21*, *Ifng*) and eosinophil-associated genes (*Epx*, *Prg2*) in lung tissues determined by qRT-PCR. (C) IFN-γ and PRG2 levels in lung tissue homogenates measured by ELISA. (D–E) Relative mRNA expression levels of Th2-related genes (*Gata3*, *Il4*) and Treg-related genes (*Foxp3*, *Il10*) in lung tissues. (F) IL-4 and IL-10 levels in lung tissue homogenates measured by ELISA. Data are presented as mean ± SEM, except for *Ifng*, PRG2, *Foxp3*, and *Il10*, which are presented as median with interquartile range (n = 5 independent animals per group). *P < 0.05, **P < 0.01, ***P < 0.001.

The Th2-associated factor *Gata3* and cytokine gene *Il4* were expressed at higher levels in Pp-14d than in Ctrl (*Gata3*, *P* < 0.01; *Il4*, *P* < 0.001; Fig 2D). Both genes were expressed at lower levels in Pp-TCBZ than in Pp-42d (*Gata3*, *P* < 0.05; *Il4*, *P* < 0.01). The Treg-associated markers *Foxp3* and *Il10* were expressed at higher levels in Pp-28d than in Ctrl (*Foxp3*, *P* < 0.01; *Il10*, *P* < 0.05; Fig 2E). Consistently, IL-4 and IL-10 concentrations were higher in Pp-14d than in Ctrl (IL-4, *P* < 0.05; IL-10, *P* < 0.001) and lower in Pp-TCBZ than in Pp-42d (both *P* < 0.01; Fig 2F). These Th2- and Treg-associated differences indicate altered host immune profiles during *P. proliferus* infection.

All ten qRT-PCR targets examined in Figs 1H, 2A, 2B, 2D, and 2E retained significant overall group effects after Benjamini–Hochberg correction (*P* < 0.001).

### 3. Changes in the TLR4/NF-κB signaling pathway during *P. proliferus* infection

We next examined the expression and phosphorylation of proteins in the canonical TLR4/NF-κB signaling pathway during *P. proliferus* infection and after treatment.

Western blot was used to compare TLR4/NF-κB pathway proteins among the experimental groups. The TLR4/β-actin ratio was higher in Pp-28d group than in Ctrl group (*P* < 0.05; Fig 3A–3B). In parallel, the p-p65/p65 ratio was higher in Pp-42d group than in Pp-14d group (*P* < 0.001) and lower in Pp-TCBZ group than in Pp-42d group (*P* < 0.01; Fig 3C). An overall group effect was also detected for the p-IκB/β-actin ratio (*P* < 0.05; Fig 3D). Together, these findings indicate stage-associated modulation of TLR4/NF-κB signaling and lower p65 phosphorylation in Pp-TCBZ than in Pp-42d.

**Fig 3:**
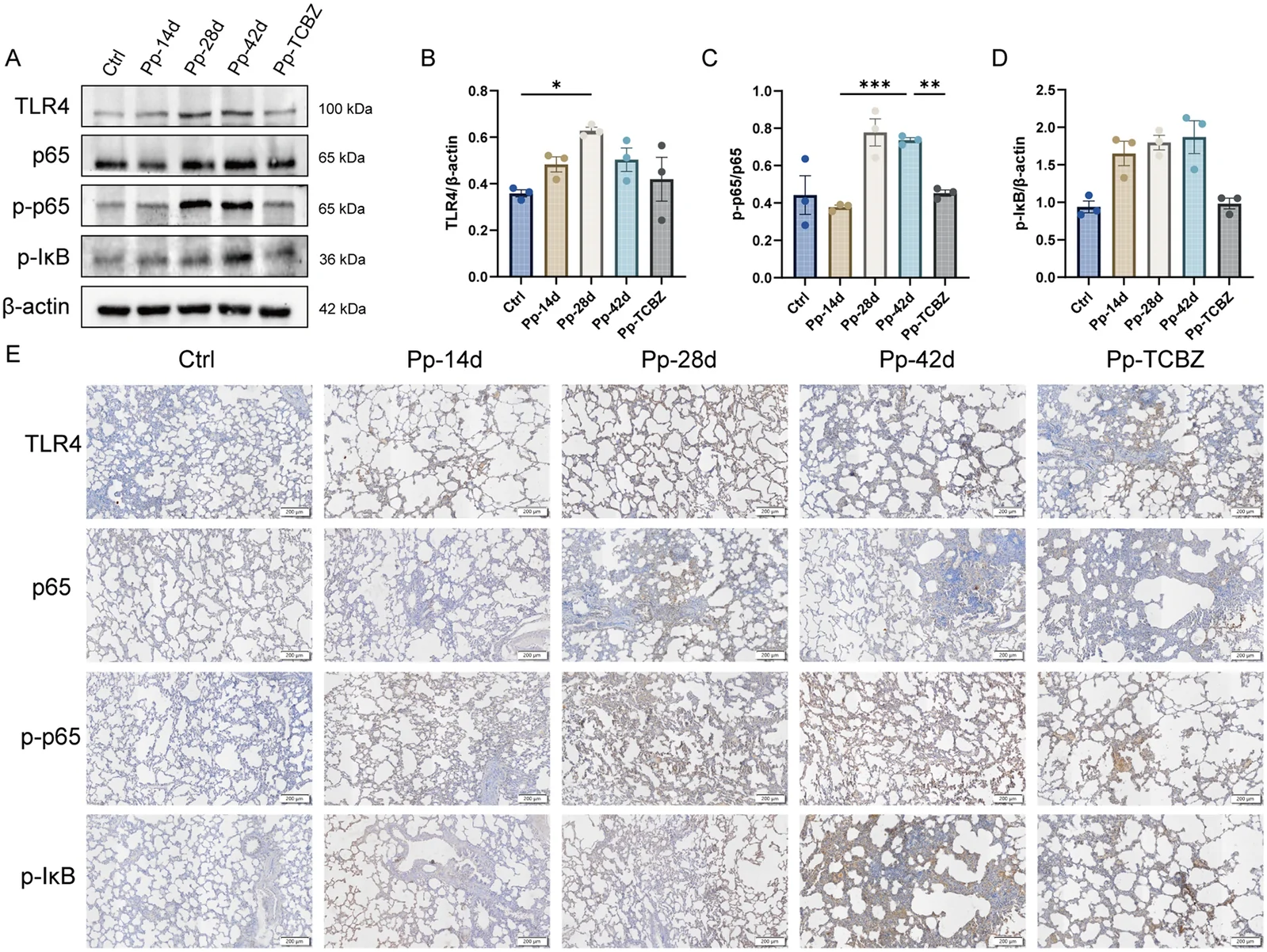
Stage-associated modulation of the pulmonary TLR4/NF-κB signaling pathway following *P. proliferus* infection and triclabendazole treatment. (A) Representative Western blot analysis of TLR4, p65, phosphorylated p65 (p-p65), phosphorylated IκB (p-IκB) and β-actin in lung tissues from different experimental groups. (B–D) Quantitative analysis of protein expression levels, including TLR4/β-actin, p-p65/p65 and p-IκB/β-actin. (E) Immunohistochemical staining of TLR4, p65, p-p65 and p-IκB in lung tissues from Ctrl, Pp-14d, Pp-28d, Pp-42d and Pp-TCBZ groups. Western blot quantification data are presented as mean ± SEM, except for the p-IκB/β-actin ratio, which is presented as median with interquartile range (n = 3 independent lung tissue samples per group). *P < 0.05, **P < 0.01, ***P < 0.001. Scale bar = 200 μm.

Consistent with the immunoblotting results, immunohistochemical staining revealed increased signals for TLR4, p65, p-p65 and p-IκB in lung tissues of infected rats (Fig 3E). Positive staining was mainly localized to inflammatory cell-rich areas and alveolar epithelial regions and appeared stronger in infected groups sampled at later post-infection time points. In contrast, staining intensity for these proteins was lower in the Pp-TCBZ group than in the Pp-42d group, further supporting lower TLR4/NF-κB pathway activation in the Pp-TCBZ group.

### 4. Stage-specific alterations in the pulmonary microbiota during *P. proliferus* infection

To compare pulmonary microbiota composition across the sampled post-infection time points and after antiparasitic treatment, we performed multi-region 5R 16S rRNA sequencing of lung tissues.

Alpha-diversity analyses showed lower pulmonary microbial richness and diversity in Pp-28d than in Ctrl (Fig 4A). The Sobs and Chao1 indices were lower in Pp-28d than in Ctrl (both *P* < 0.01) and were also lower in Pp-42d than in Ctrl (both *P* < 0.05). Shannon diversity was lower in Pp-28d than in Ctrl (*P* < 0.001), whereas the Simpson index was higher in Pp-28d than in Ctrl (*P* < 0.01) and Pp-42d (*P* < 0.05).

**Fig 4:**
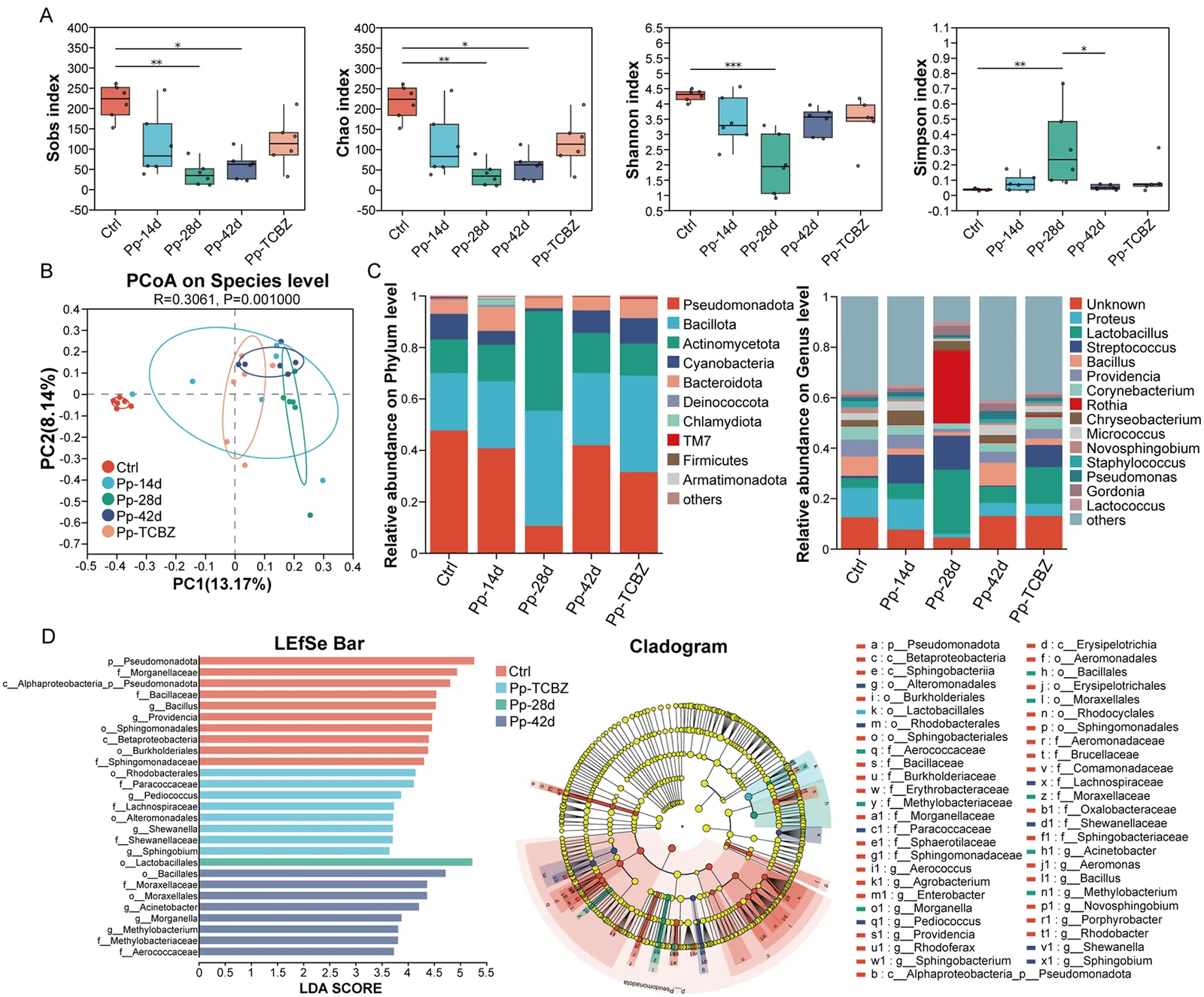
Pulmonary microbial community alterations across *P. proliferus* infection stages and after triclabendazole treatment. (A) Alpha diversity of the pulmonary microbiota in control, infected and triclabendazole-treated rats, assessed using the Sobs, Chao1, Shannon and Simpson indices. (B) Principal coordinates analysis (PCoA) based on Bray–Curtis dissimilarities illustrating differences in pulmonary microbial community structure among experimental groups. (C) Relative abundance of pulmonary microbiota at the phylum and genus levels across different infection stages and following triclabendazole treatment. (D) Linear discriminant analysis effect size (LEfSe) identifying bacterial taxa differentially represented among experimental groups. The left panel shows taxa identified based on LDA scores, and the right panel presents the corresponding cladogram. An LDA score threshold of 3.5 was applied. For the alpha-diversity indices in panel A, box plots show the median and interquartile range (*n* = 6 per group), and each dot represents one animal. *P < 0.05, **P < 0.01, ***P < 0.001.

Community-level differences were further evaluated using Bray–Curtis dissimilarities. Species-level PCoA showed significant group-wise separation of the pulmonary microbiota (*P* < 0.01; Fig 4B). The Pp-14d group showed marked deviation from the Ctrl profile. The Pp-28d and Pp-42d groups showed community profiles that differed from Pp-14d while remaining distinct from Ctrl. The Pp-TCBZ group exhibited a microbial community profile closer to that of Ctrl.

Taxonomic profiling showed group-specific relative-abundance patterns at the phylum and genus levels (Fig 4C). Across the five groups, the dominant phyla included Pseudomonadota, Bacillota, Actinomycetota, Cyanobacteria, and Bacteroidota. Compared with the other groups, Pp-28d showed a lower relative abundance of Pseudomonadota and higher relative abundances of Bacillota and Actinomycetota. At the genus level, the top taxa included *Proteus*, *Lactobacillus*, *Streptococcus*, *Bacillus*, *Providencia*, *Corynebacterium*, *Rothia*, *Chryseobacterium*, *Micrococcus* and *Novosphingobium*, among which *Lactobacillus* was present in all five groups and reached its highest relative abundance in Pp-28d. The genus-level profile of Pp-TCBZ appeared more similar to that of Ctrl than to that of Pp-42d.

Linear discriminant analysis effect size (LEfSe) identified taxa differentially represented among the experimental groups (Fig 4D). The lungs in the Ctrl group were characterized by enrichment of multiple lineages, including the families of Bacillaceae, Morganellaceae and Sphingomonadaceae, the genus *Providencia*. LEfSe analysis identified distinct taxonomic signatures across groups. In the Ctrl group, Pseudomonadota and related taxa contributed most to the microbial composition, whereas in the Pp-28d group, Lactobacillales was the dominant discriminant order, reflecting the pronounced increase of *Lactobacillus* at the genus level. In the Pp-42d group, LEfSe highlighted enrichment of the families of Moraxellaceae and Aerococcaceae, the order Moraxellales and the genera of *Acinetobacter* and *Methylobacterium*, together with additional discriminant lineages such as Methylobacteriaceae. In the post-treatment group, discriminant features included the order Rhodobacterales, the family of Lachnospiraceae and the genera of *Pediococcus* and *Shewanella* (Fig 4D).

Collectively, multi-region 5R profiling revealed group-level differences in pulmonary microbiota composition, including separation of the infected groups from Ctrl, compositional differences among the Pp-14d, Pp-28d and Pp-42d groups, and distinct microbial signatures in Pp-TCBZ compared with Pp-42d.

### 5. Spatial distribution of Lactobacillus-associated signals in the lungs during *P. proliferus* infection

To further investigate the spatial distribution of pulmonary bacteria, particularly *Lactobacillus*, FISH was performed on lung tissue sections (Fig 5A). *Lactobacillus*-associated signals were detected in lung tissues from infected groups, particularly in lesion-associated regions. The white arrow in Fig 5A indicates a representative signal.

**Fig 5:**
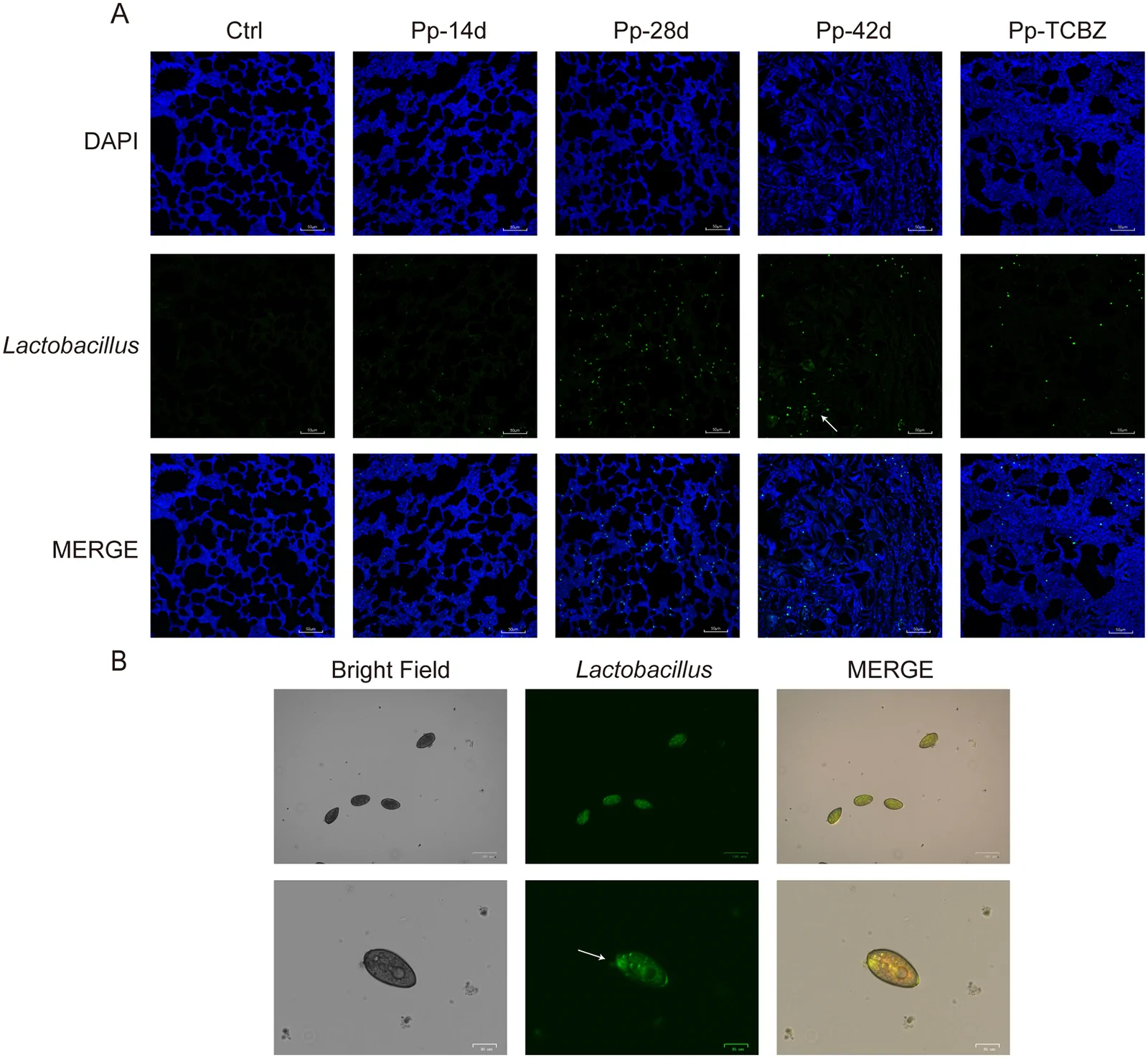
Fluorescence *in situ* hybridization (FISH) detection of bacterial distribution in lung tissue and parasite eggs. (A) Representative FISH images showing bacterial localization in lung tissues from different groups. Nuclei were stained with DAPI (blue). *Lactobacillus* was detected using a Cy5-labeled specific probe, and the Cy5 signal was pseudocolored green for visualization. Merged images show the localization of *Lactobacillus*-associated signals. The white arrow indicates a representative signal. Scale bar = 50 μm. (B) FISH detection of bacteria associated with parasite eggs. Bright-field images show isolated parasite eggs, whereas fluorescence images show *Lactobacillus*-associated signals detected using a Cy5-labeled specific probe and pseudocolored green for visualization. Merged images demonstrate the spatial association of these signals with parasite eggs. The white arrow indicates a representative signal on the egg surface. Scale bar = 100 μm; enlarged view scale bar = 35 μm.

*Lactobacillus*-specific staining of isolated *P. proliferus* eggs showed *Lactobacillus*-associated signals on the egg surface after release from the adult worm (Fig 5B). The white arrow in Fig 5B indicates a representative *Lactobacillus*-associated signal on the egg surface. Bright-field images confirmed egg morphology. These observations suggest that *Lactobacillus*-associated signals can be detected on parasite eggs after their release from the worm.

## Discussion

Pulmonary injury differed across the defined post-infection sampling time points. Histopathological analyses showed greater inflammatory infiltration and alveolar architectural disruption in infected groups sampled at later post-infection time points, with the most extensive collagen deposition observed in the Pp-42d group (Fig 1C–1G). These pathological features are consistent with the typical life history of *P. proliferus* infection, which involves destructive migration and cyst formation in tissues [2,21]. Compared with untreated infected rats sampled at 42 dpi, triclabendazole-treated rats showed no detectable worms and less severe inflammatory injury, although lung architecture was not fully restored.

However, because the Pp-TCBZ group was sampled at 60 dpi, the differences between these groups may reflect a combination of parasite clearance, treatment-related effects, and the longer recovery interval.

Helminth infections commonly induce type 2 and regulatory immune responses that can limit tissue damage while facilitating parasite persistence [22,23]. In the present study, we examined pulmonary immune changes associated with *P. proliferus* infection. Our results showed that group-level differences in innate immune signaling were accompanied by differences in adaptive immune-associated profiles. Gene expression analyses showed higher *Ifng* and *Tbx21* expression in earlier sampled infected groups, whereas *Il4* and *Il10* expression was higher in later sampled infected groups. This group-level pattern suggests stronger Th1/eosinophil-associated activation in earlier sampled infected groups and stronger Th2- and regulatory T cell (Treg)-associated features in later sampled infected groups (Fig 2). This pattern is typical of helminth infections, where type-2 immune responses help with tissue repair while also allowing the parasite to survive [24]. Macrophage activation driven by IL-4Rα signaling has been shown to coordinate wound healing and fibrotic remodeling in helminth-associated lung inflammation [25], providing a potential explanation for the fibrosis observed in our model. The increased IL-10 levels indicate the activation of regulatory pathways that mitigate excessive inflammatory damage, a characteristic of numerous chronic helminth infections [23]. Together, these molecular profiles suggest a Th2/Treg-associated immune environment that may support parasite persistence. Similar type 2 and regulatory immune biases have been described across helminth infections, where IL-4- and IL-10-associated responses can suppress inflammatory immunity and favor parasite persistence [22,26].

In our previous studies, we found that *P. proliferus* could transport gut microbiota to the lungs and establish stable colonization, forming a new pulmonary microbiota [16]. The TLR4/NF-κB signaling axis is a core innate immune mechanism for host defense against bacterial invasion [27] and it may also play a role in counteracting parasites [28]. Based on this, we assessed the TLR4/NF-κB signaling axis. The TLR4/β-actin ratio was higher in Pp-28d than in Ctrl, whereas the p-p65/p65 ratio was higher in Pp-42d than in Pp-14d and Pp-TCBZ (Fig 3). These group-level observations suggest that both migratory injury and later parasite residency may contribute to innate immune stimulation. Comparable involvement of innate immune pathways has been described in other helminth infections [29].

Our previous study used V3–V4 16S rRNA sequencing to identify infection-associated microbiota differences between infected and uninfected rats [16]. The present study extends that work by applying multi-region 5R 16S rRNA sequencing [30] to assess group-level microbiome differences across fixed post-infection sampling time points and after triclabendazole treatment, together with FISH localization. Unlike the previous study, which primarily compared infected and uninfected animals, the present work further evaluated microbiome differences among multiple post-infection sampling groups and after antiparasitic treatment, providing additional information regarding group-level microbial variation associated with different experimental conditions. Pulmonary microbiota composition differed among the sampled groups. *Rothia*, *Streptococcus* and *Lactobacillus* were more abundant at 28 dpi, whereas *Acinetobacter*-related lineages were more prominent at 42 dpi (Fig 4). Fluorescence *in situ* hybridization detected bacterial rRNA signals in lesion-associated regions of infected lung tissue, showing bacterial presence in inflamed areas (Fig 5A). These changes suggest that parasite-induced tissue injury may create microenvironments that support microbial persistence or expansion, possibly through epithelial barrier disruption or altered immune regulation [31]. This finding implies that pulmonary infection may reshape the local microbial ecosystem in parallel with host immune responses.

Group-level differences in pulmonary microbiota were observed alongside group-level variation in TLR4/NF-κB signaling. Changes in bacterial community composition may therefore have contributed to innate immune activation, although this relationship was not tested directly. Notably, several taxa that exhibited increased abundance during infection, such as those within the *Lactobacillus* genus, have been documented to possess immunomodulatory or anti-inflammatory properties within mucosal tissues [32,33]. These bacteria are recognized for their capacity to modulate cytokine production and epithelial immune signaling pathways. Consequently, in the setting of parasite-induced lung injury, the higher representation of these taxa may reflect a compensatory microbial response associated with the IL-10-related regulatory immune environment observed in infected lungs [23]. Although the present data cannot establish causality, these observations suggest a possible association between pulmonary microbiota composition and inflammatory regulation during *P. proliferus* infection. However, helminth-associated microbiome findings have not been uniform across studies: experimental *Necator americanus* infection produced no detectable changes in overall gut microbial community structure or diversity, while a meta-analysis indicated that microbiome effects may vary according to helminth species and anatomical niche [34,35].

Bacterial associations with helminths have been described in several parasite systems. Depletion of the endosymbiont *Wolbachia* inhibited embryogenesis in female *Onchocerca volvulus* [36], whereas bacterial contact promoted the hatching of *Trichuris muris* eggs [37]. Pan-bacterial FISH has also detected 16S rRNA signals in multiple developmental stages of *Schistosoma mansoni*, including eggs [38]. These findings provide a comparative context for the *Lactobacillus*-associated signals observed on isolated *P. proliferus* eggs in the present study. Beyond the group-level differences in pulmonary microbiota, FISH showed co-localization of *Lactobacillus*-associated signals with isolated *P. proliferus* eggs (Fig 5B), suggesting a possible spatial association. Whether this association is biologically specific remains to be determined. Many *Lactobacillus* species are renowned for their potent immunomodulatory and anti-inflammatory properties at mucosal surfaces, particularly their capacity to induce Treg expansion and IL-10 production [39,40]. These observations raise the possibility that egg-associated *Lactobacillus* may influence local immune recognition through mechanisms involving IL-10 production and Treg responses. Whether such immunomodulation occurs remains to be determined, as the observed co-localization could alternatively reflect nonspecific bacterial adherence to the egg surface.

These findings collectively indicate group-level differences in the pulmonary pathophysiological environment during *P. proliferus* infection. Pulmonary fibrosis, innate immune signaling, Th2/Treg-associated immune features and pulmonary microbiota composition differed among groups sampled at defined post-infection time points and after treatment. Compared with untreated infected rats at 42 dpi, triclabendazole-treated rats showed lower parasite burdens and weaker inflammatory responses. These findings provide a basis for further investigation of pulmonary immune and microbial changes and tissue recovery after antiparasitic treatment.

Several limitations should be considered when interpreting our findings. The repeated cross-sectional design supports comparisons among groups sampled at fixed post-infection time points but does not capture within-animal trajectories over time. Although housing conditions were standardized across groups, potential cage-related effects on the microbiome outcomes cannot be entirely excluded. The modest group sizes may limit the precision of estimates for smaller effects, particularly for secondary and exploratory outcomes; effect sizes and 95% confidence intervals are therefore reported to support interpretation of the findings. Only male SD rats were used in this study, consistent with commonly used experimental designs for *Paragonimus* infection models. Although this approach facilitated comparison with previous studies, sex-dependent differences in pulmonary immunity and microbiome composition may influence host responses, and validation in female animals will be required in future studies. In addition, although SD rats are not confirmed natural definitive hosts of *P. proliferus*, they provide a practical experimental model because they support parasite development and allow controlled investigation of pulmonary pathological and immunological responses. Finally, metacercariae used in this study were collected from a single endemic region, and validation using parasite populations from different geographic origins will be needed to determine the broader applicability of these findings.

## Conclusion

In summary, using a repeated cross-sectional rat model of lung-stage *P. proliferus* infection, we identified group-level pulmonary microenvironment differences across fixed post-infection sampling time points, including more severe fibrosis in later sampled infected groups, differences in Th1/eosinophil- and Th2/Treg-associated immune profiles, and stage-associated modulation of the TLR4/NF-κB signaling axis. Furthermore, we observed stage-associated group-level differences in the pulmonary microbiome, together with *Lactobacillus*-associated signals on isolated parasite eggs, suggesting a possible spatial association. These findings characterize group-level associations among parasite development, immune profiles and pulmonary microbial ecology, providing a framework for further investigation of host–parasite–microbiota interactions in pulmonary paragonimiasis.

## Supporting information

S1 Appendix Supplementary Methods, detailed statistical analysis and bioinformatic processing of multi-region 16S rRNA sequencing data; Table A, ATS lung injury histopathological scoring criteria; Table B, qRT-PCR primer sequences; Table C, primer sequences used for multi-region 5R 16S rRNA gene amplification; Table D, outcome classification and statistical analysis plan; Table E, assumption testing and statistical procedures; Table F, omnibus statistical results, effect sizes, Benjamini–Hochberg FDR correction, and post hoc power analysis for the primary outcomes; Table G, post hoc pairwise comparisons and multiplicity-adjusted *P* values; Table H, sequencing depth and species-level diversity quality metrics; Fig A, representative H&E-stained lung fields illustrating the item-level scoring categories; Fig B, rarefaction curves for species-level alpha-diversity indices. (DOCX)

S1 Raw images Original uncropped and unadjusted Western blot images underlying Fig 3. (PDF)

S1 Data Original LEfSe output generated using an LDA score threshold >3.5. (CSV)

S1 Checklist ARRIVE Guidelines 2.0 author checklist. (PDF)
