# Supplementary material for: Stage-specific remodeling of the pulmonary microenvironment during Paragonimus proliferus infection in a rat model
Source: PLoS Negl Trop Dis. 2026 Sep 25;20(9):e0014760. doi: 10.1371/journal.pntd.0014760 (PMC13626465; doi:10.1371/journal.pntd.0014760)

Original uncropped and unadjusted Western blot images underlying Fig 3A. Blots 1–3 represent three independent biological samples per group. For each blot, the lane order after the molecular-weight marker is Ctrl, Pp-14d, Pp-28d, Pp-42d, and Pp- TCBZ.

TLR4: Lane order after the molecular-weight marker: Ctrl, Pp-14d, Pp-28d, Pp-42d, Pp-TCBZ.

Blot 1:

100kDa
70kDa

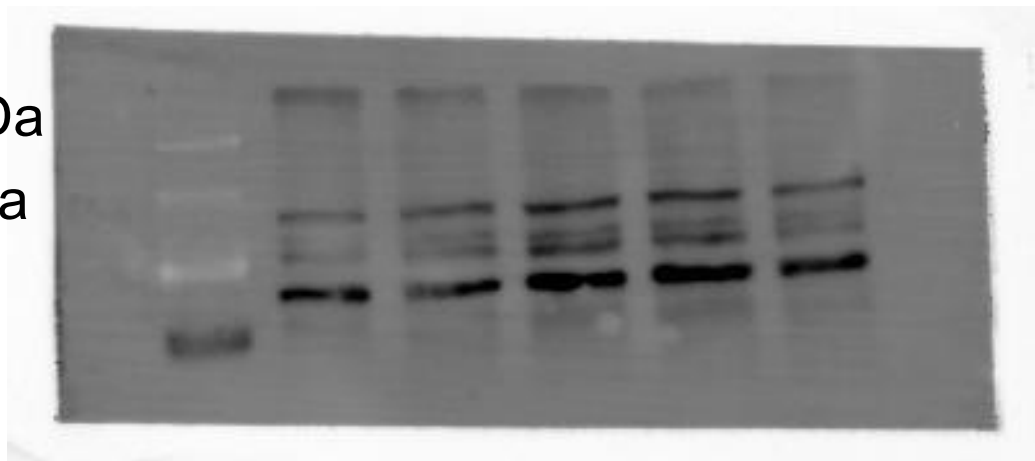

Used in Fig 3A

Blot 2:

100kDa
70kDa

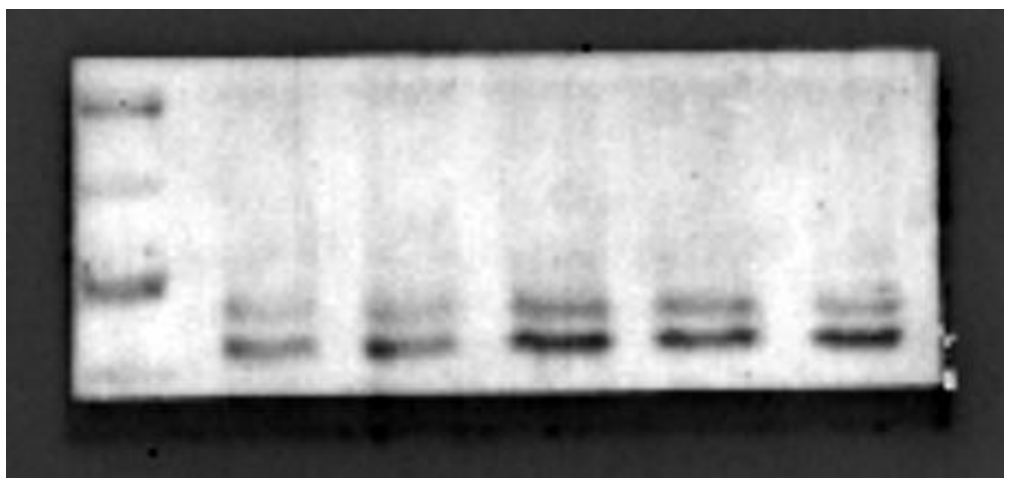

Blot 3:

100kDa
70kDa

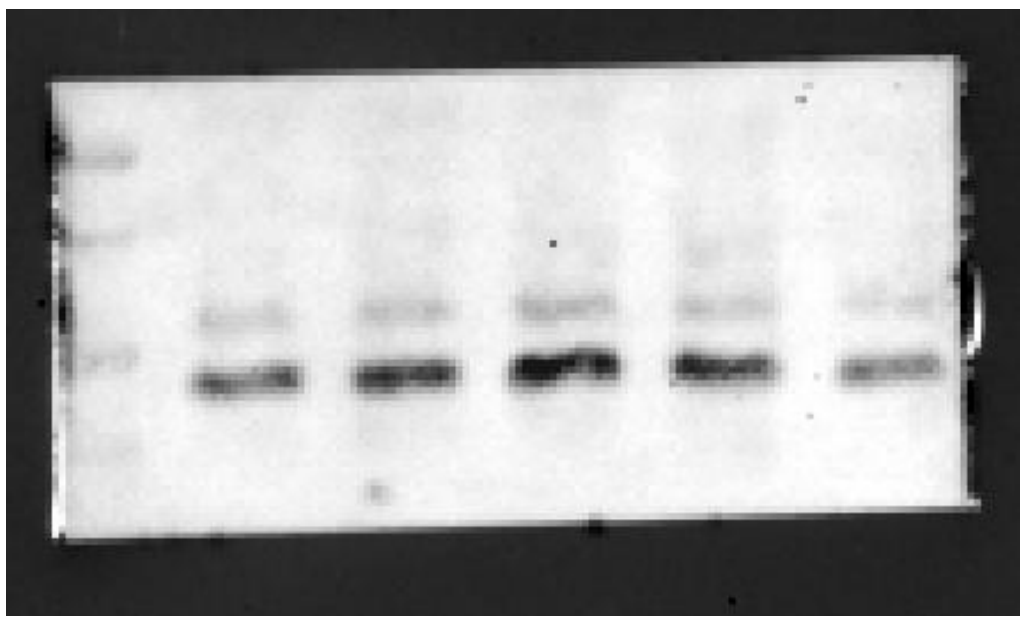

β-actin: Lane order after the molecular-weight marker: Ctrl, Pp-14d, Pp-28d, Pp-42d, Pp-TCBZ.

Blot 1:

40kDa
35kDa

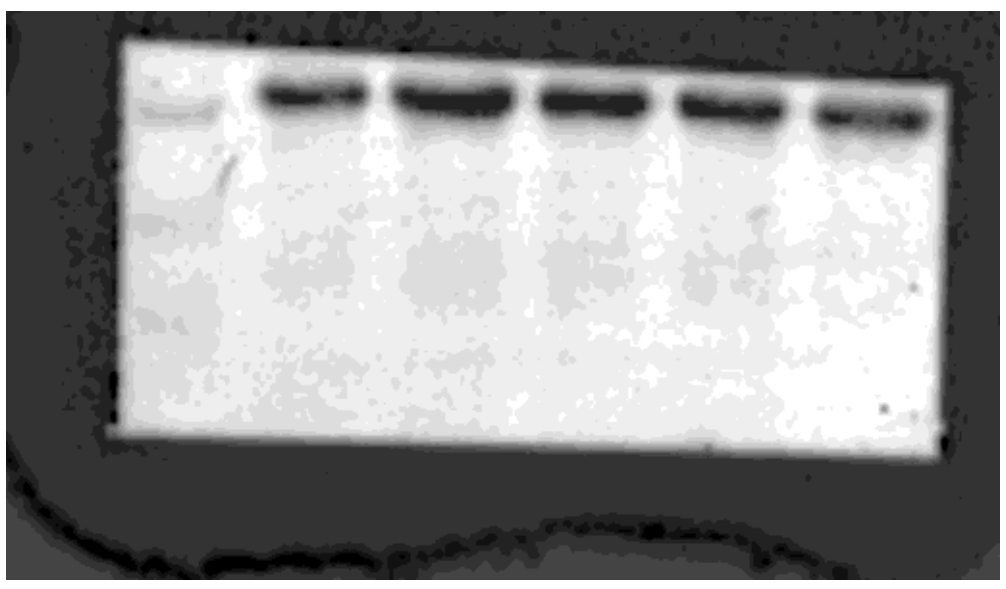

Blot 2:

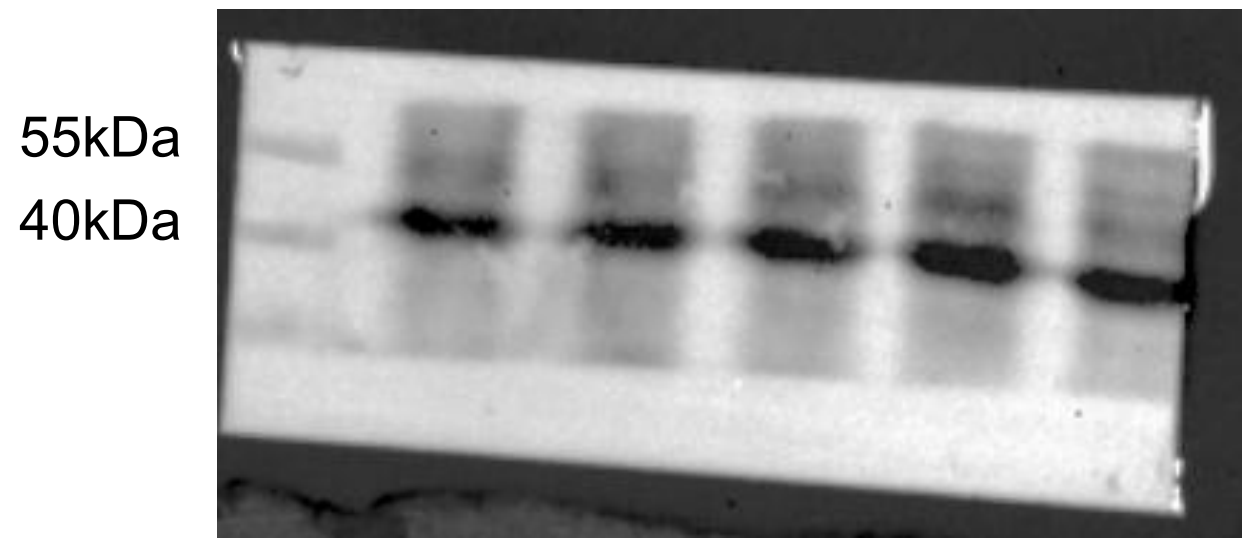

Blot 3:

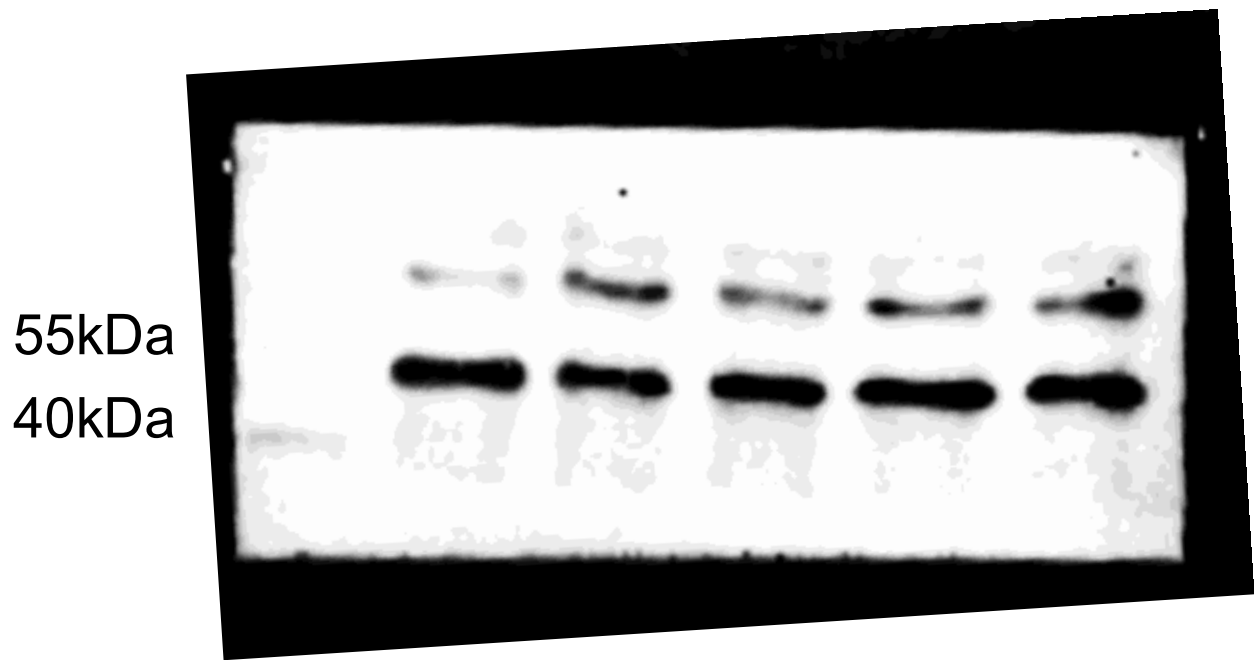

p65: Lane order after the molecular-weight marker: Ctrl, Pp-14d, Pp-28d, Pp-42d, Pp-TCBZ.

Blot 1:

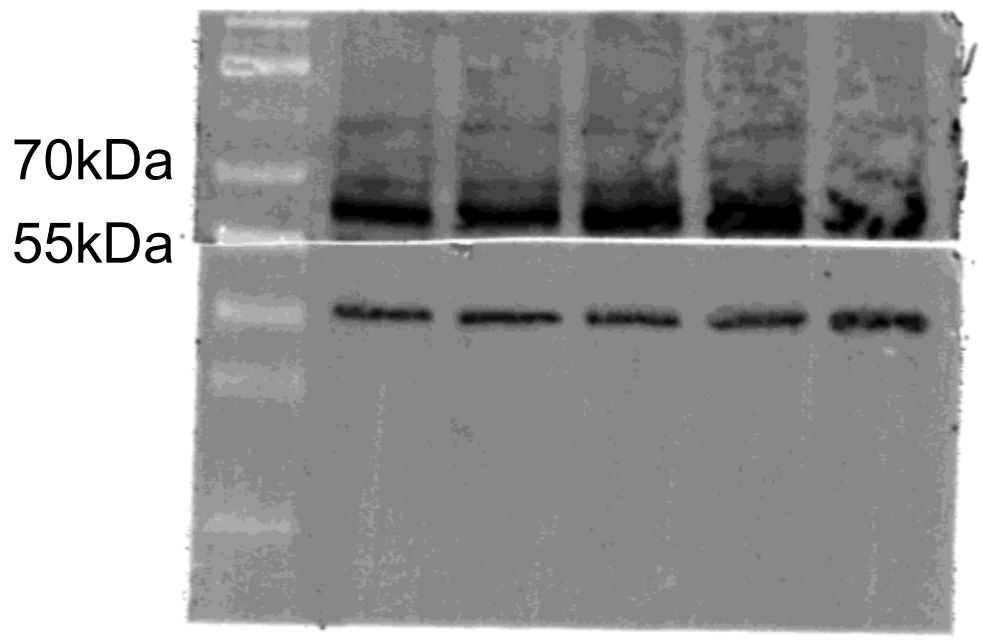

Upper: p65

Blot 2:

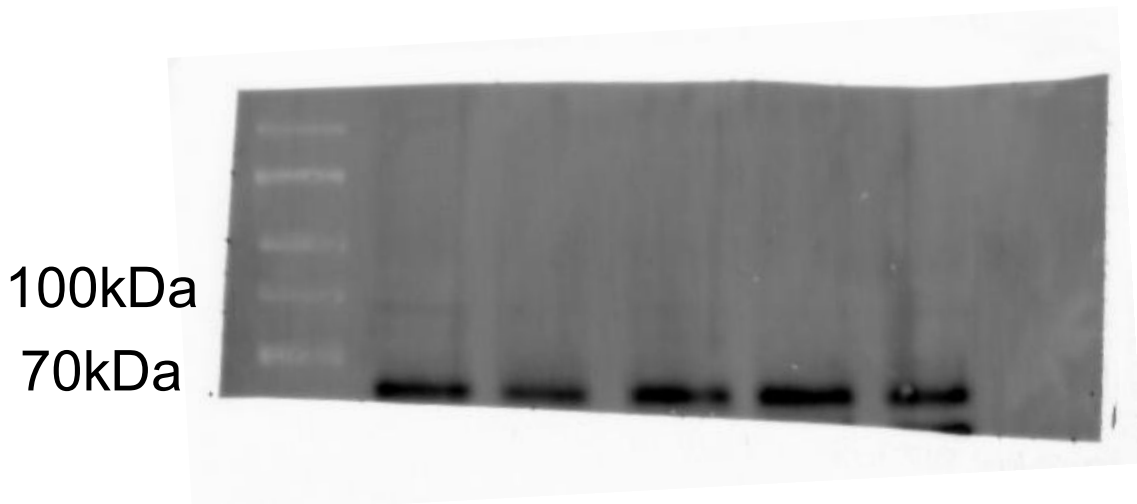

Used in Fig 3A

Blot 3:

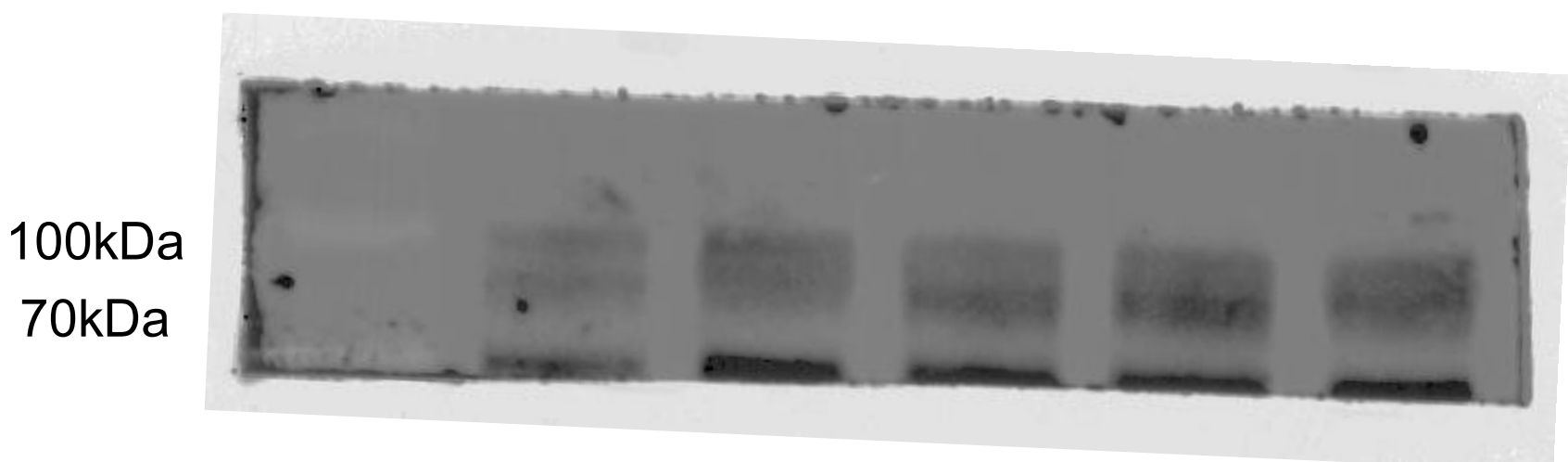

β-actin: Lane order after the molecular-weight marker: Ctrl, Pp-14d, Pp-28d, Pp-42d, Pp-TCBZ.

Blot 1:

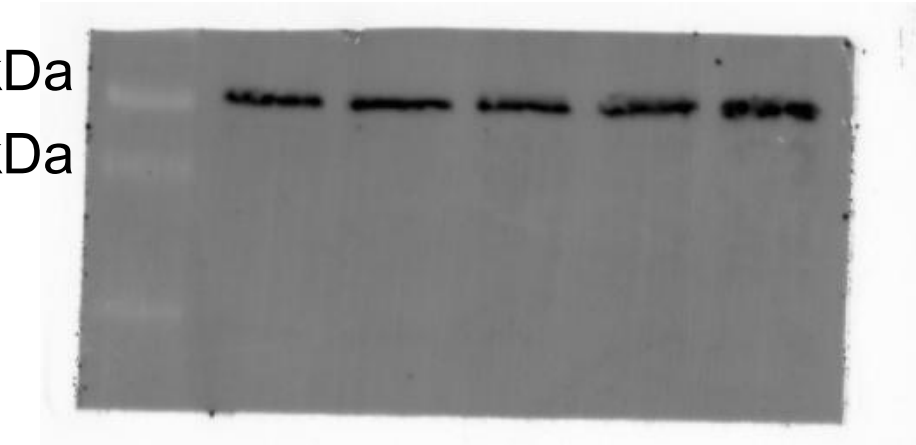

Blot 2:

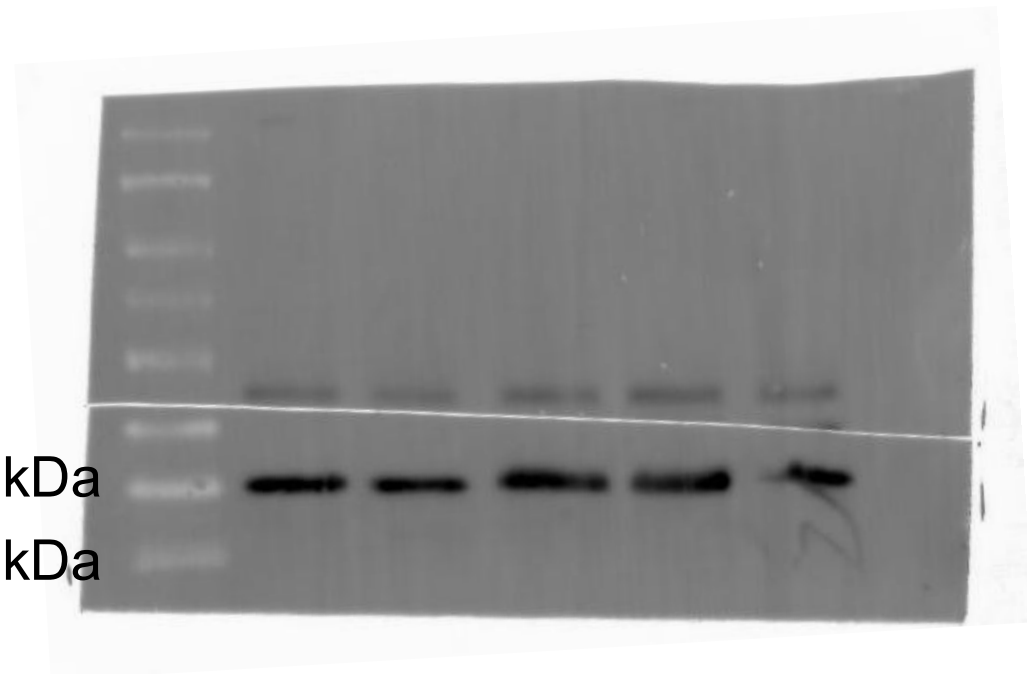

Blot 3:

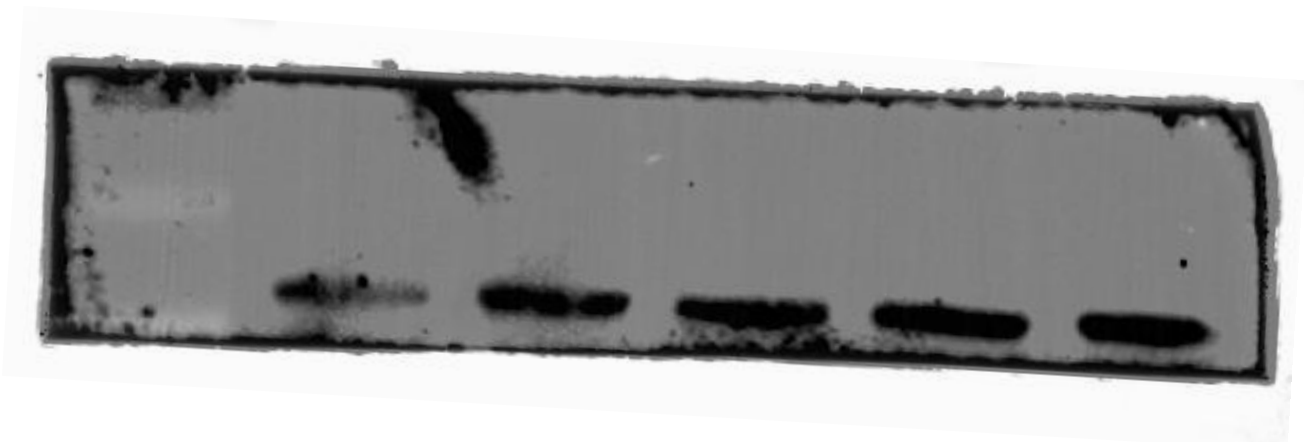

p-p65: Lane order after the molecular-weight marker: Ctrl, Pp-14d, Pp-28d, Pp-42d, Pp-TCBZ.

Blot 1:

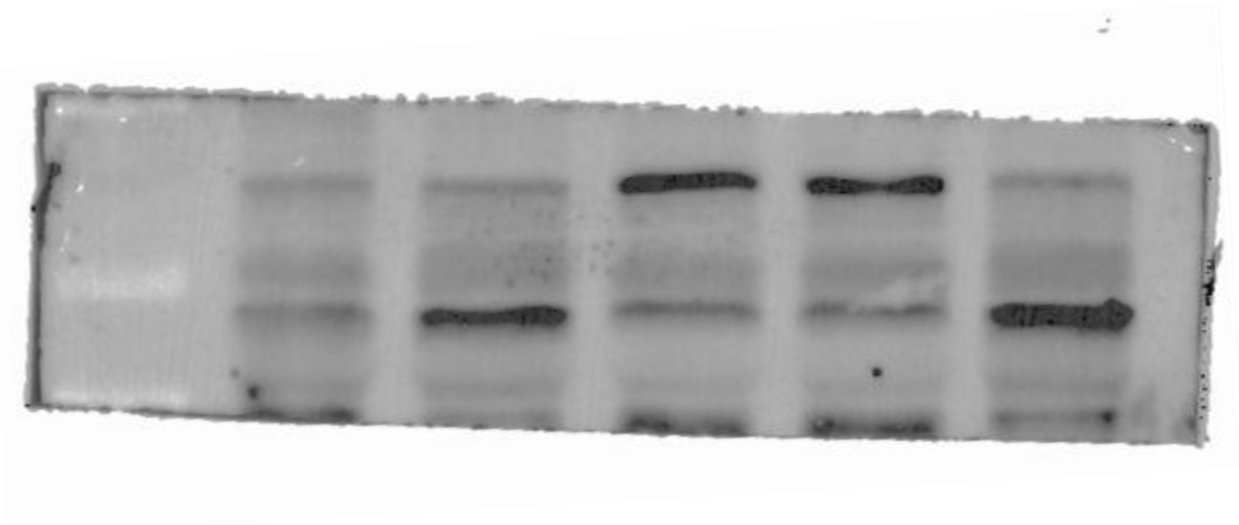

Used in Fig 3A

Blot 2:

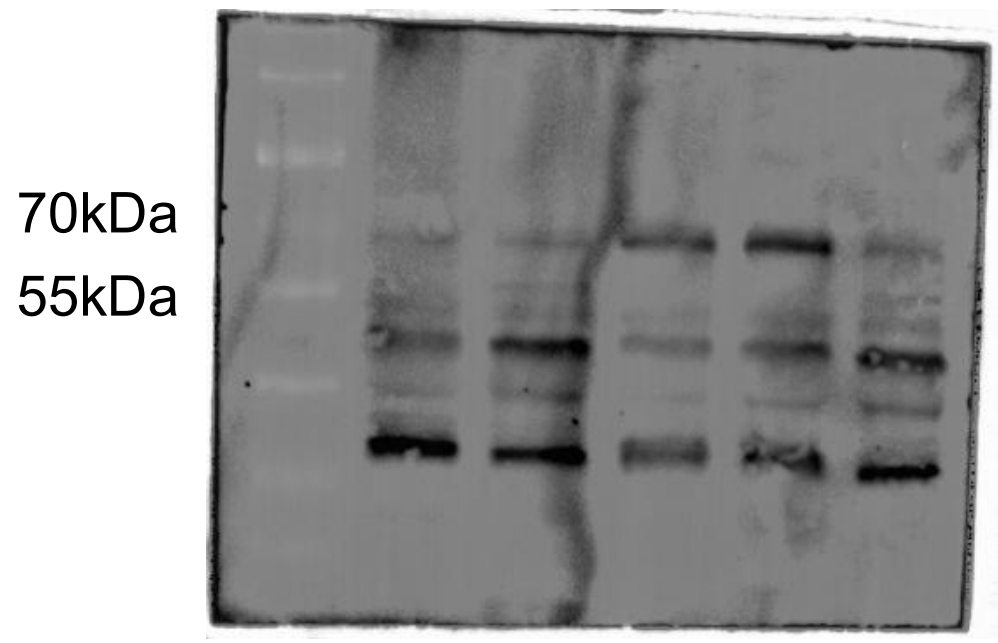

Blot 3:

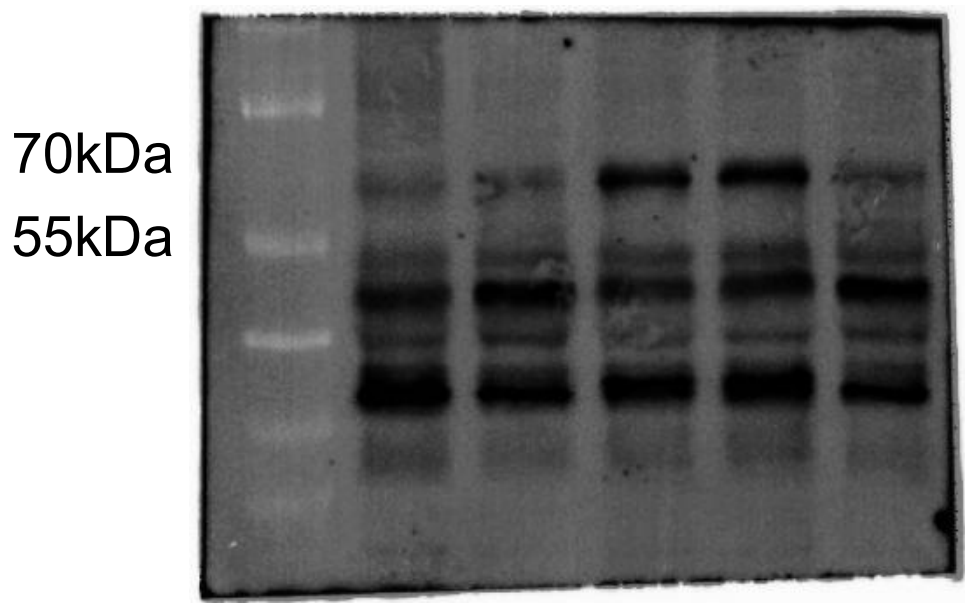

β-actin: Lane order after the molecular-weight marker: Ctrl, Pp-14d, Pp-28d, Pp-42d, Pp-TCBZ.

Blot 1:

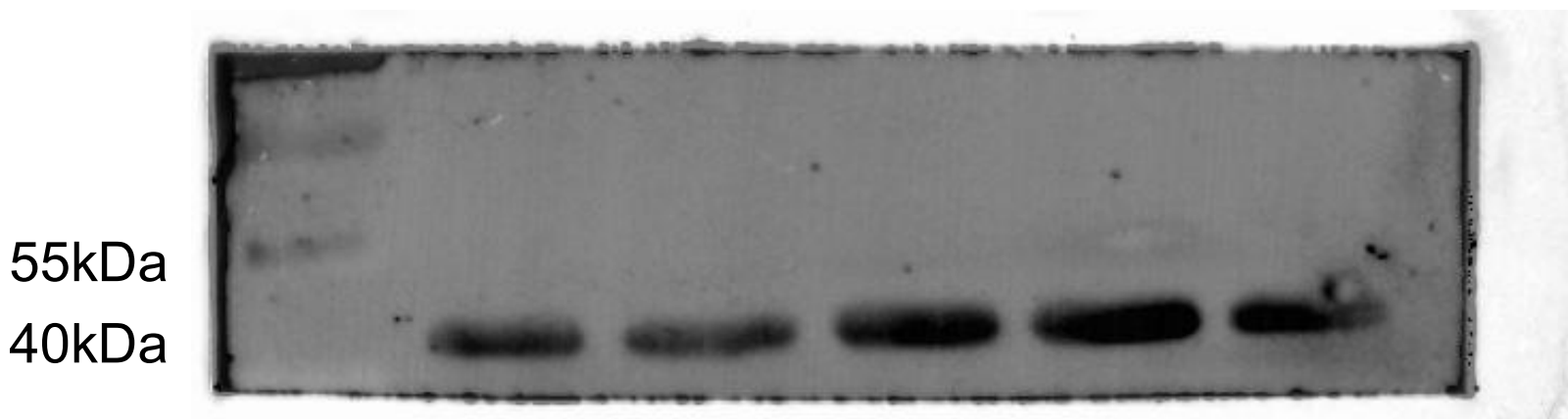

Blot 2:

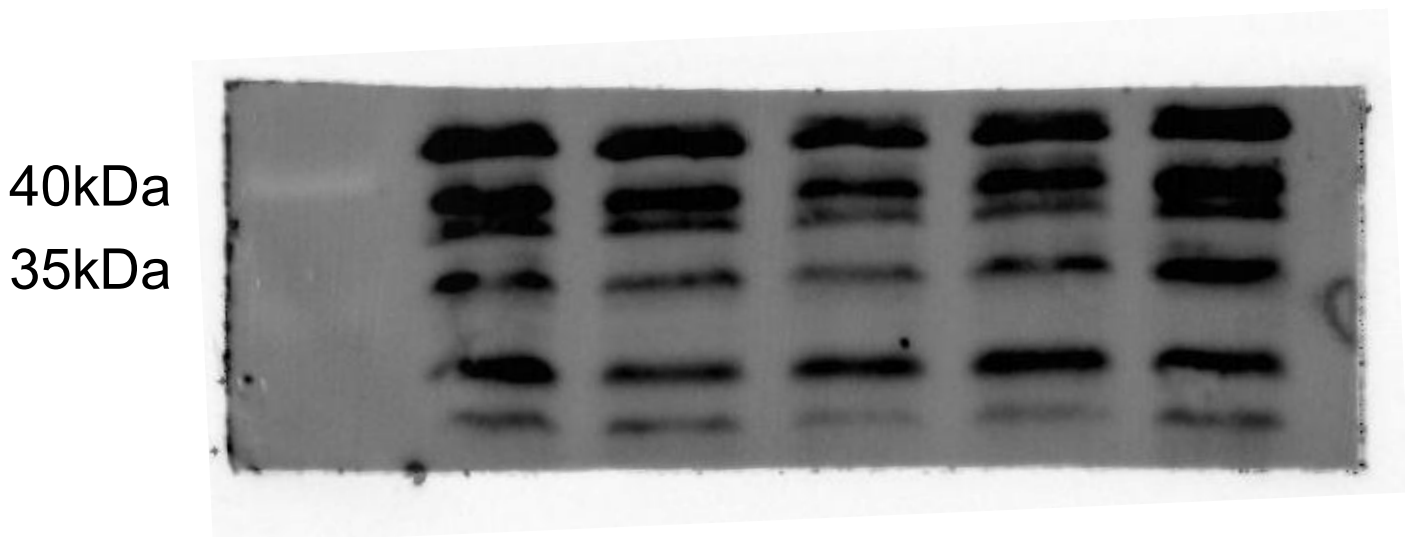

Blot 3:

55kDa
40kDa

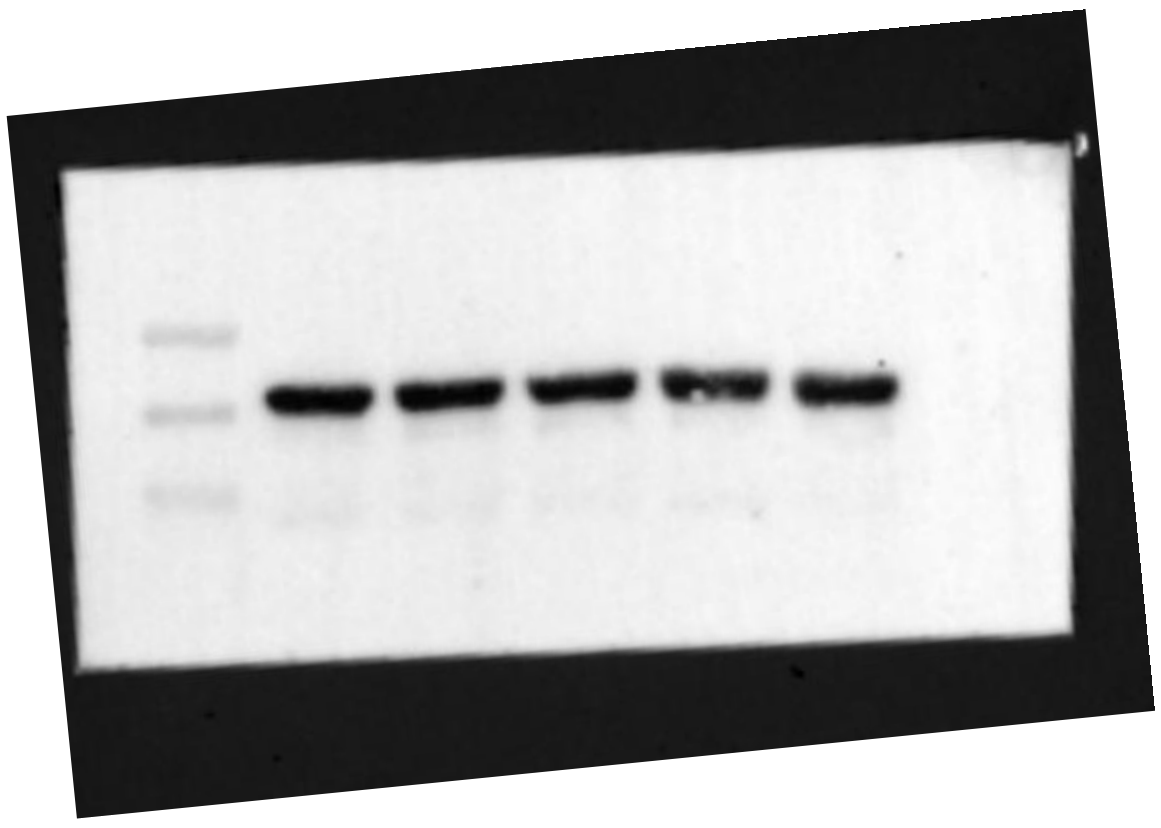

Used in Fig 3A

p-IκB: Lane order after the molecular-weight marker: Ctrl, Pp-14d, Pp-28d, Pp-42d, Pp-TCBZ.

Blot 1:

40kDa
35kDa

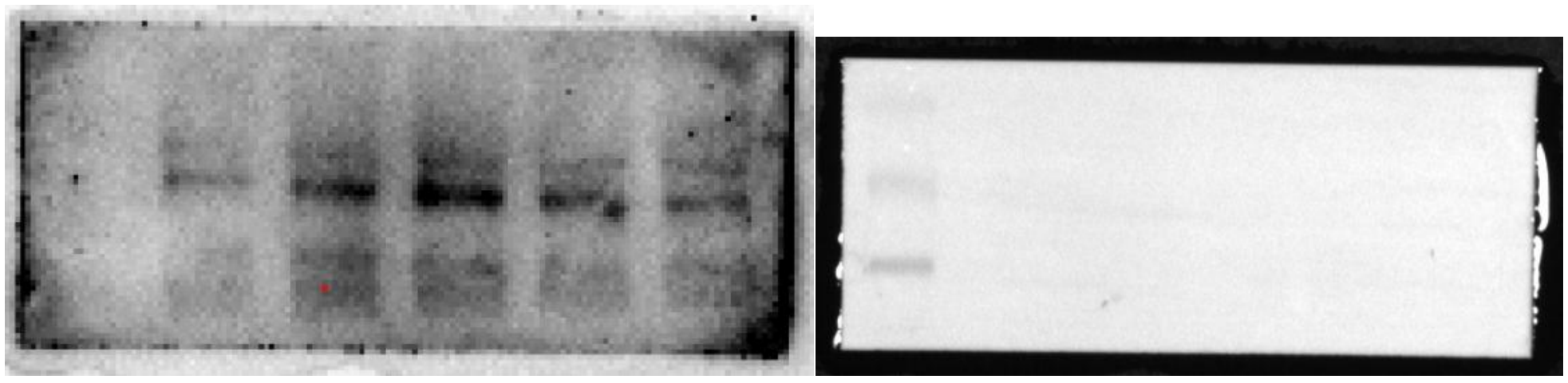

Blot 2:

40kDa
35kDa

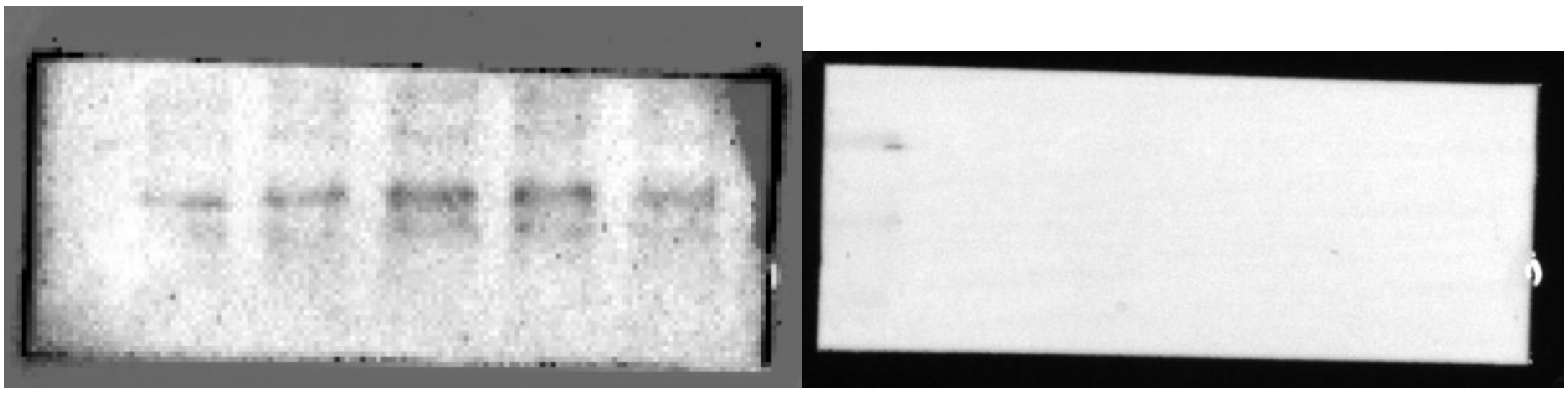

Blot 3:

40kDa
35kDa

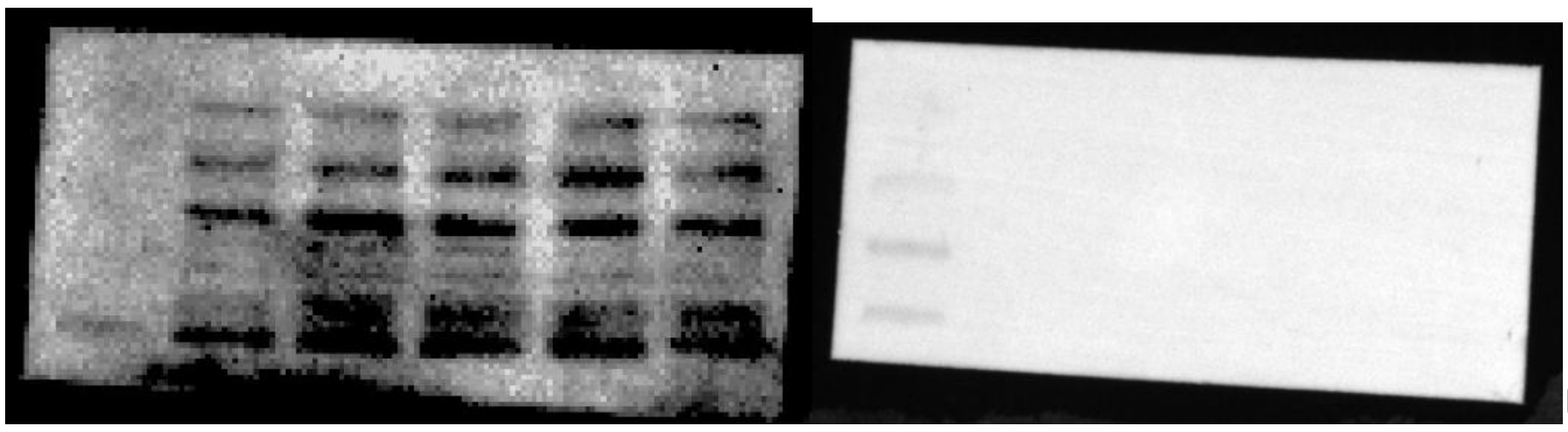

Used in Fig 3A

β-actin: Lane order after the molecular-weight marker: Ctrl, Pp-14d, Pp-28d, Pp-42d, Pp-TCBZ.

Blot 1:

55kDa
40kDa

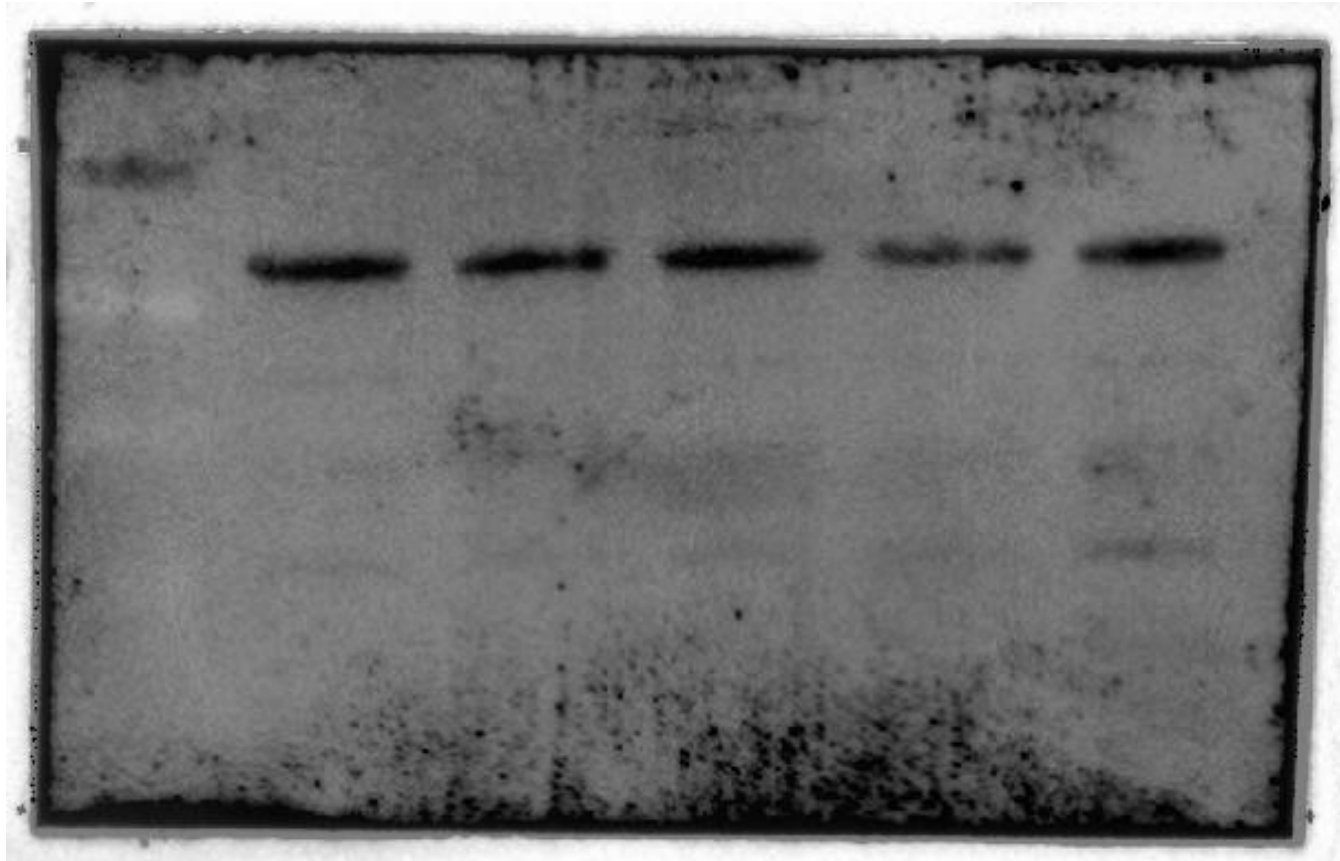

Blot 2:

55kDa
40kDa

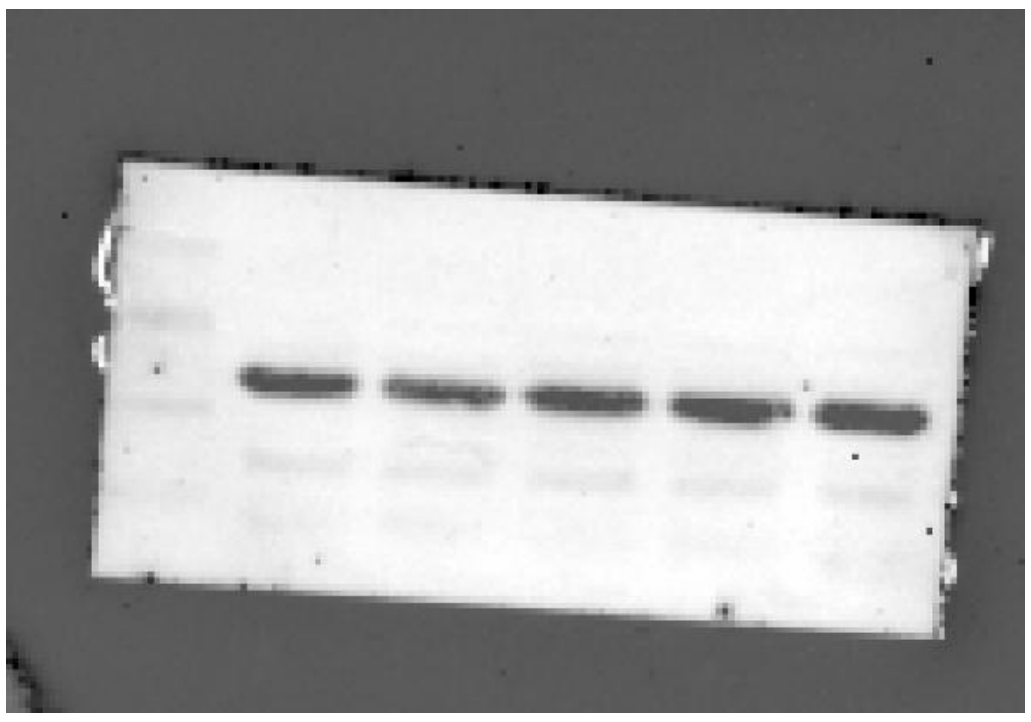

Blot 3:

55kDa
40kDa

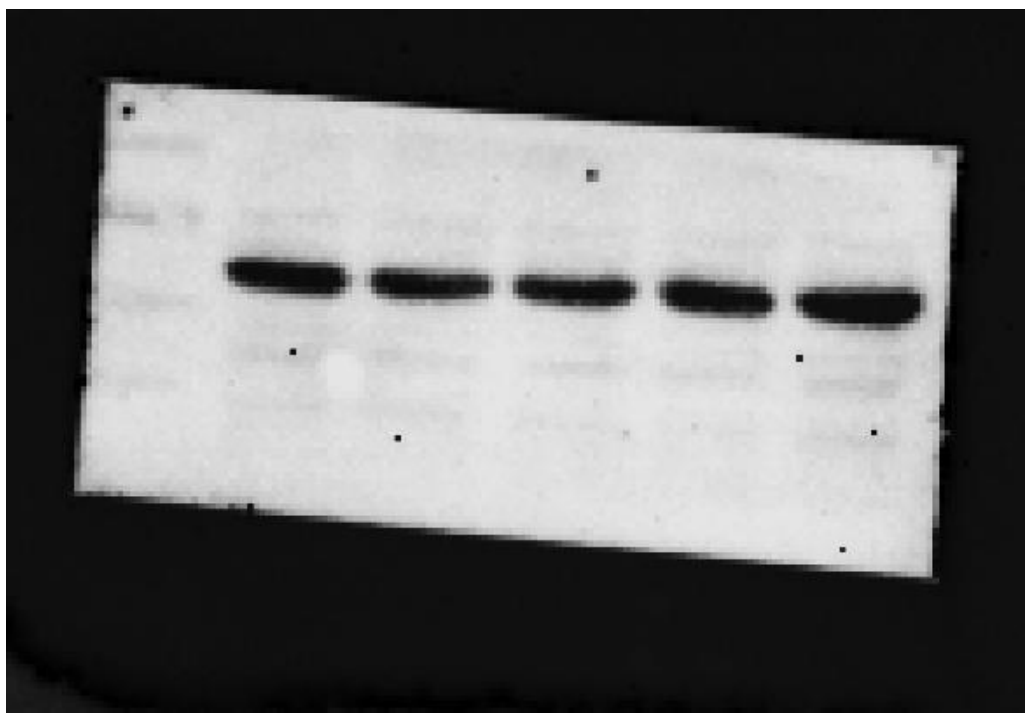

Supplement: Supplementary file 1 [file pntd.0014760.s002.pdf]
