# Supplementary material for: Stage-specific remodeling of the pulmonary microenvironment during Paragonimus proliferus infection in a rat model
Source: PLoS Negl Trop Dis. 2026 Sep 25;20(9):e0014760. doi: 10.1371/journal.pntd.0014760 (PMC13626465; doi:10.1371/journal.pntd.0014760)
Supplement: Supplementary file 2 [file pntd.0014760.s004.pdf]

The ARRIVE guidelines 2.0: author checklist

The ARRIVE Essential 10

These items are the basic minimum to include in a manuscript. Without this information, readers and reviewers cannot assess the reliability of the findings.

Item		Recommendation	Section/line number, or reason for not reporting
**Study design**	1	For each experiment, provide brief details of study design including:	
		a. The groups being compared, including control groups. If no control group has been used, the rationale should be stated.	Materials and Methods, Experimental Design and Antiparasitic Treatment ; Fig 1A. Five groups were compared: Ctrl, Pp-14d, Pp-28d, Pp-42d, and Pp-TCBZ.
		b. The experimental unit (e.g. a single animal, litter, or cage of animals).	Materials and Methods, Statistical Analysis. Each animal was considered the experimental unit and an independent biological replicate.
**Sample size**	2	a. Specify the exact number of experimental units allocated to each group, and the total number in each experiment. Also indicate the total number of animals used.	legends. Thirty rats were allocated to five groups (six rats per group). Six animals per group were used for microbiome profiling, five per group for histopathology, qRT-PCR, and ELISA, and three independent lung
		b. Explain how the sample size was decided. Provide details of any *a priori* sample size calculation, if done.	Table F. No formal a priori sample-size calculation was performed. Group sizes were selected on the basis of feasibility and the principle of minimizing animal use; post hoc achieved power for the primary outcomes is
**Inclusion and exclusion criteria**	3	a. Describe any criteria used for including and excluding animals (or experimental units) during the experiment, and data points during the analysis. Specify if these criteria were established *a priori*. If no criteria were set, state this explicitly.	Materials and Methods, Animals and Experimental Infection. Eligibility was based on the specified strain, sex, age, body-weight range, and completion of acclimatization. No additional a priori exclusion criteria were specified
		b. For each experimental group, report any animals, experimental units or data points not included in the analysis and explain why. If there were no exclusions, state so.	No animals, experimental units, or data points were excluded from the analyses.
		c. For each analysis, report the exact value of *n* in each experimental group.	Materials and Methods, Experimental Design and Antiparasitic Treatment ; individual figure legends. The exact value of n for each analysis and experimental group is reported.
**Randomisation**	4	a. State whether randomisation was used to allocate experimental units to control and treatment groups. If done, provide the method used to generate the randomisation sequence.	Materials and Methods, Experimental Design and Antiparasitic Treatment. Rats were randomly assigned to the five experimental groups before infection; the specific method used to generate the allocation sequence was not documented.
		b. Describe the strategy used to minimise potential confounders such as the order of treatments and measurements, or animal/cage location. If confounders were not controlled, state this explicitly.	Experimental Infection and Experimental Design and Antiparasitic Treatment. Cage specifications, housing conditions, bedding, chow, water, and replacement schedules were standardized across groups to minimize environmental confounding. No additional control of treatment or measurement order
**Blinding**	5	Describe who was aware of the group allocation at the different stages of the experiment (during the allocation, the conduct of the experiment, the outcome assessment, and the data analysis).	Materials and Methods, Histological Staining and Analysis. Histopathological fields were scored independently by two observers blinded to group allocation. Personnel conducting the remaining experimental procedures and analyses were aware of group allocation.
**Outcome measures**	6	a. Clearly define all outcome measures assessed (e.g. cell death, molecular markers, or behavioural changes).	Introduction, final paragraph; Materials and Methods, individual experimental subsections; S1 Appendix, Table D. Primary, secondary, and exploratory outcome measures are defined.
		b. For hypothesis-testing studies, specify the primary outcome measure, i.e. the outcome measure that was used to determine the sample size.	Introduction, final paragraph; S1 Appendix, Table D. Relative ATS lung injury score and collagen area fraction were designated as the primary outcome domains. No formal a priori sample-size calculation based on a single primary outcome was performed
**Statistical methods**	7	a. Provide details of the statistical methods used for each analysis, including software used.	Analysis ; S1 Appendix, Supplementary Methods and Tables D-G. Statistical analyses were performed using GraphPad Prism 10.4.1 and R. The statistical tests and corresponding multiple-comparison procedures used for each analysis are
		b. Describe any methods used to assess whether the data met the assumptions of the statistical approach, and what was done if the assumptions were not met.	Analysis ; S1 Appendix, Supplementary Methods and Table E. Normality and homogeneity of variance were assessed using the Shapiro-Wilk and Levene s tests. Ordinary one-way ANOVA, Welch s ANOVA, or the Kruskal-Wallis test was
**Experimental animals**	8	a. Provide species-appropriate details of the animals used, including species, strain and substrain, sex, age or developmental stage, and, if relevant, weight.	Materials and Methods, Animals and Experimental Infection. Male Sprague-Dawley rats, aged 8-12 weeks and weighing 275 ± 25 g, were used.
		b. Provide further relevant information on the provenance of animals, health/immune status, genetic modification status, genotype, and any previous procedures.	Materials and Methods, Animals and Experimental Infection. Rats were obtained from the Experimental Animal Center of Kunming Medical University and maintained under specific pathogen-free conditions.
**Experimental procedures**	9	For each experimental group, including controls, describe the procedures in enough detail to allow others to replicate them, including:	
		a. What was done, how it was done and what was used.	Experimental Infection, Experimental Design and Antiparasitic Treatment, Sample Collection, Tissue Processing and Preservation, and the individual experimental-method subsections. The
		b. When and how often.	Design and Antiparasitic Treatment. Infection, sampling time points, triclabendazole dose, treatment frequency, treatment duration, and post-treatment
		c. Where (including detail of any acclimatisation periods).	Experimental Infection and Sample Collection, Tissue Processing and Preservation. The source and housing of the animals, the 14-day acclimatization period, and the locations and conditions of
		d. Why (provide rationale for procedures).	Methods, Animals and Experimental Infection and Experimental Design and Antiparasitic Treatment. The rationale for the animal model, study groups, sampling time points, and treatment design is
**Results**	10	For each experiment conducted, including independent replications, report:	
		a. Summary/descriptive statistics for each experimental group, with a measure of variability where applicable (e.g. mean and SD, or median and range).	Appendix, Tables F-H. Summary statistics and measures of variability are reported for the experimental groups. Normally distributed data are presented as mean ± SEM, and non-normally distributed data as
		b. If applicable, the effect size with a confidence interval.	S1 Appendix, Tables F-G. Effect sizes and 95% confidence intervals are reported for the applicable statistical analyses.

The Recommended Set

These items complement the Essential 10 and add important context to the study. Reporting the items in both sets represents best practice.

Item		Recommendation	Section/line number, or reason for not reporting
**Abstract**	11	Provide an accurate summary of the research objectives, animal species, strain and sex, key methods, principal findings, and study conclusions.	Abstract. The research objectives, animal mode
**Background**	12	a. Include sufficient scientific background to understand the rationale and context for the study, and explain the experimental approach.	Introduction. The scientific background, rationale, and experimental approach are described.
		b. Explain how the animal species and model used address the scientific objectives and, where appropriate, the relevance to human biology.	Introduction; Materials and Methods, Animals and Experimental Infection. The suitability of the Sprague-Dawley rat model for studying P. proliferus infection and its relevance to the study objectives are described.
**Objectives**	13	Clearly describe the research question, research objectives and, where appropriate, specific hypotheses being tested.	Introduction, final paragraph. The research obje
**Ethical statement**	14	Provide the name of the ethical review committee or equivalent that has approved the use of animals in this study, and any relevant licence or protocol numbers (if applicable). If ethical approval was not sought or granted, provide a justification.	Materials and Methods, Ethics Statement. Animal experiments were approved by the Animal Ethics Committee of Kunming Medical University (Approval No. KMMUX2025070214).
**Housing and husbandry**	15	Provide details of housing and husbandry conditions, including any environmental enrichment.	Materials and Methods, Animals and Experin
**Animal care and monitoring**	16	a. Describe any interventions or steps taken in the experimental protocols to reduce pain, suffering and distress.	Experimental Infection and Sample Collection, Tissue Processing and Preservation. Animals were monitored every 2 days, with food and water replenished and bedding changed as needed. Anesthesia and euthanasia procedures are
		b. Report any expected or unexpected adverse events.	No expected or unexpected adverse events were observed.
		c. Describe the humane endpoints established for the study, the signs that were monitored and the frequency of monitoring. If the study did not have humane endpoints, state this.	intake, and signs of respiratory distress. Humane endpoints included persistent severe distress, marked inactivity, inability to eat or drink, or severe respiratory difficulty. Animals meeting any of these criteria would have
**Interpretation/ scientific implications**	17	a. Interpret the results, taking into account the study objectives and hypotheses, current theory and other relevant studies in the literature.	Discussion. The findings are interpreted in relation to the study objectives, current knowledge, and relevant published studies.
		b. Comment on the study limitations including potential sources of bias, limitations of the animal model, and imprecision associated with the results.	limitations should be considered when interpreting our findings. Potential sources of bias, limitations of the repeated cross-sectional design and animal model, cage-related effects, group sizes, and
**Generalisability/ translation**	18	Comment on whether, and how, the findings of this study are likely to generalise to other species or experimental conditions, including any relevance to human biology (where appropriate).	Discussion, paragraph beginning Several limitations should be considered when interpreting our findings. Generalisability is discussed in relation to the use of male rats, the status of Sprague-Dawley rats as a non-confirmed natural definitive host, and the use of metacercariae from a single endemic region.
**Protocol registration**	19	Provide a statement indicating whether a protocol (including the research question, key design features, and analysis plan) was prepared before the study, and if and where this protocol was registered.	A study protocol was prepared before the experiment as part of the ethical review process, but it was not registered in a public repository.
**Data access**	20	Provide a statement describing if and where study data are available.	Data Availability Statement; Supporting Informa
**Declaration of interests**	21	a. Declare any potential conflicts of interest, including financial and non-financial. If none exist, this should be stated.	Competing interests. The authors have declared that no competing interests exist.
		b. List all funding sources (including grant identifier) and the role of the funder(s) in the design, analysis and reporting of the study.	Funding. All funding sources, grant identifiers, recipients, and the role of the funders are reported. The funders had no role in study design, data collection and analysis, the decision to publish, or preparation of the manuscript.
