# Supplementary material for: Stage-specific remodeling of the pulmonary microenvironment during Paragonimus proliferus infection in a rat model
Source: PLoS Negl Trop Dis. 2026 Sep 25;20(9):e0014760. doi: 10.1371/journal.pntd.0014760 (PMC13626465; doi:10.1371/journal.pntd.0014760)
Supplement: Supplementary file 3 [file pntd.0014760.s001.docx]

**Supplementary Methods:**

**Bioinformatic processing of multi-region 16S rRNA sequencing data**

Raw reads were quality-filtered using fastp (v0.19.6), followed by sequence denoising and ASV inference using the DADA2 plugin in QIIME 2 (release 2024). Region-specific read counts from the V2, V3, V5, V6 and V8 regions were integrated using SMURF with default settings. Taxonomic assignment was performed using the classify-sklearn Naive Bayes classifier against the SILVA 138.2 16S rRNA database, with a confidence threshold of 0.7. Three negative controls were processed in parallel with the biological samples and submitted to the same sequencing workflow, and environmental samples were submitted in parallel for background monitoring. No PCR amplification products were obtained from any of the three negative controls; consequently, no sequence reads or microbial taxa were recovered from these controls. Because the negative controls contained no detectable microbial features, no features were removed from the biological dataset on the basis of negative-control screening, and no model-based decontamination analysis was performed. For downstream diversity analyses, the sequence table was rarefied to 124,225 sequences per sample, corresponding to the minimum denoised sequence count among the 30 analyzed samples.

**Detailed statistical analysis**

No prospective power calculation was performed because directly comparable effect-size estimates for the prespecified primary outcomes were unavailable. Sample sizes were determined by experimental feasibility and the ethical principle of minimizing animal use. Five animals per group were analyzed for histopathology, qRT-PCR, and ELISA outcomes, three independent lung tissue samples per group were analyzed by Western blotting, and six animals per group were included in pulmonary microbiome profiling. Pulmonary pathological injury and fibrosis were prespecified as the primary outcome domains and were assessed using the relative ATS lung injury score and collagen area fraction (CAF), respectively. qRT-PCR, ELISA, and TLR4/NF-κB pathway outcomes were classified as secondary, whereas pulmonary microbiome outcomes were considered exploratory (Table D).

For each continuous outcome, normality was assessed within each experimental group using the Shapiro–Wilk test, and homogeneity of variance was assessed using Levene’s test. Outcomes satisfying both assumptions were analyzed using ordinary one-way ANOVA followed by Tukey’s multiple-comparisons test. Normally distributed outcomes with unequal variances were analyzed using Welch’s one-way ANOVA followed by the Games–Howell test. Outcomes that did not satisfy the normality assumption were analyzed using the Kruskal–Wallis test followed by Dunn’s multiple-comparisons test (Table E). For qRT-PCR data, statistical inference was based on ΔCt values, whereas 2^−ΔΔCt values are presented for visualization. Global effect sizes were reported as η² for parametric analyses and ε² for Kruskal–Wallis analyses. Corresponding 95% percentile bootstrap confidence intervals were calculated using 10,000 within-group resamples. Post hoc achieved power for the two prespecified primary outcomes was estimated using a fixed-effects omnibus one-way ANOVA model with five groups, five animals per group, and α = 0.05 (Table F).

The Benjamini–Hochberg procedure was applied separately within the histopathology, qRT-PCR, ELISA, TLR4/NF-κB pathway, and pulmonary microbiota alpha-diversity outcome families. Within the qRT-PCR family, correction was applied across the omnibus *P* values for the ten target genes. Pairwise comparisons were conducted only after a significant omnibus test and were adjusted using the corresponding multiple-comparisons procedure. Beta diversity was evaluated using a single global ANOSIM test. Taxonomic profiles and LEfSe features were treated as exploratory results and were not included in the FDR-controlled outcome families. Exact omnibus and pairwise results, including raw and adjusted *P* values, effect sizes, and confidence intervals, are provided in Tables F and G.

**Table A. ATS lung injury histopathological scoring criteria**

| Parameter | 0 | 1 | 2 |
| --- | --- | --- | --- |
| A. Neutrophils in the alveolar space | None | 1–5 | >5 |
| B. Neutrophils in the interstitial space | None | 1–5 | >5 |
| C. Hyaline membranes | None | 1 | >1 |
| D. Proteinaceous debris filling the airspaces | None | 1 | >1 |
| E. Alveolar septal thickening | <2× | 2–4× | >4× |

Note: Score = [(20*A)+(14*B)+(7*C)+(7*D)+(2*E)]/(number of fields*100), score range 0 to 1.

**Table B. qRT-PCR primer sequences**

| **Species** | **Target gene** | | **Forward primer (5′–3′)** | **Reverse primer (5′–3′)** |
| --- | --- | --- | --- | --- |
| Rat | ***Il1b*** | AATCTCACAGCAGCATCTCGACAAG | | TCCACGGGCAAGACATAGGTAGC |
| Rat | ***Tnfa*** | CCACCACGCTCTTCTGTCTA | | AGGGTCTGGGCCATAGAACT |
| Rat | ***Ifng*** | GGCAAAAGGACGGTAACACG | | TTCACCTCGAACTTGGCGAT |
| Rat | ***Tbx21*** | CACTGGATGCCGACAGGAAGT | | CCGTCATTCACCTCCACGAT |
| Rat | ***Il4*** | CGTGATGTACCTCCGTGCTT | | GTGAGTTCAGACCGCTGACA |
| Rat | ***Gata3*** | ACCAGGAGATCATGTGACCC | | CTCAAGCCAGGAGTTTGCCA |
| Rat | ***Foxp3*** | CCATAATATGCGGCCCCCTT | | GCGGGTGGTTTCTGAAGTA |
| Rat | ***Il10*** | GCTCAGCACTGCTATGTTGC | | TTGTCACCCCGGATGGAATG |
| Rat | ***Epx*** | AACCAGTTCAGAAGAGCCCG | | TTGTCGCACACAATCCGAGA |
| Rat | ***Prg2*** | CAAAGCTCAGTTGGTCTGCC | | CCCTGGTTGATTCCCCTGAC |
| Rat | ***Gapdh*** | AAGTTCAACGGCACAGTCAAGG | | GACATACTCAGCACCAGCATCAC |

**Table C. Primer sequences used for multi-region 5R 16S rRNA gene amplification**

| **Target region** | **Direction** | **Primer sequence (5′–3′)** |
| --- | --- | --- |
| V2 | Forward | TGGCGAACGGGTGAGTAA |
|  | Reverse | CCGTGTCTCAGTCCCARTG |
| V3 | Forward | ACTCCTACGGGAGGCAGC |
|  | Reverse | GTATTACCGCGGCTGCTG |
| V5 | Forward | GTGTAGCGGTGRAATGCG |
|  | Reverse | CCCGTCAATTCMTTTGAGTT |
| V6 | Forward | GGAGCATGTGGWTTAATTCGA |
|  | Reverse | CGTTGCGGGACTTAACCC |
| V8 | Forward | GGAGGAAGGTGGGGATGAC |
|  | Reverse | AAGGCCCGGGAACGTATT |

**Table D. Outcome classification and statistical analysis plan**

| **Outcome family** | **Outcomes** | **Classification** | **Primary null hypothesis / analysis** |
| --- | --- | --- | --- |
| Histopathology | Relative ATS lung injury score, CAF | Primary | No difference among Ctrl, Pp-14d, Pp-28d, Pp-42d and Pp-TCBZ |
| qPCR genes | *Il1b*, *Tnfa*, *Tbx21*, *Ifng*, *Epx*, *Prg2*, *Gata3*, *Il4*, *Foxp3*, *Il10* | Secondary | Group differences tested after assumption checking |
| Cytokines / ELISA proteins | IFN-γ, PRG2, IL-4, IL-10 | Secondary | Group differences tested after assumption checking |
| TLR4/NF-κB pathway proteins | TLR4, p-p65/p65, p-IκB/β-actin | Secondary | Group differences tested after assumption checking |
| Alpha diversity | Sobs, Chao1, Shannon, Simpson | Exploratory | Group differences tested after assumption checking |
| Beta diversity | Bray–Curtis PCoA and global ANOSIM | Exploratory | Overall pulmonary microbial community composition does not differ among the five groups; single global ANOSIM test |
| Microbiome taxonomic comparisons | Phylum/genus-level taxa, LEfSe features | Exploratory | Relative-abundance profiling and LEfSe analysis (no FDR correction) |

**Table E. Assumption testing and statistical procedures for outcomes with available individual-level data**

| **Figure panel** | **Outcome** | **Shapiro–Wilk *P* by group** | **Levene’s test *P*** | **Statistical procedure** |
| --- | --- | --- | --- | --- |
| Figure 1F | Relative ATS lung injury score | 0.9101 / 0.9807 / 0.9478 / 0.9642 / 0.9470 | 0.7895 | One-way ANOVA + Tukey |
| Figure 1G | Collagen area fraction (CAF) | 0.1443 / 0.5368 / 0.6004 / 0.9994 / 0.3774 | 0.1704 | One-way ANOVA + Tukey |
| Figure 1H | *Il1b* | 0.5031 / 0.8707 / 0.3783 / 0.1502 / 0.1752 | 0.0054 | Welch’s one-way ANOVA + Games–Howell |
|  | *Tnfa* | 0.4345 / 0.2189 / 0.3485 / 0.4972 / 0.7670 | 0.2852 | One-way ANOVA + Tukey |
| Figure 2A | *Tbx21* | 0.3018 / 0.8515 / 0.7132 / 0.1731 / 0.4580 | 0.2857 | One-way ANOVA + Tukey |
|  | *Ifng* | 0.0228 / 0.0250 / 0.6770 / 0.6840 / 0.4971 | 0.4732 | Kruskal–Wallis + Dunn |
| Figure 2B | *Epx* | 0.2463 / 0.5110 / 0.5903 / 0.6194 / 0.0804 | 0.0585 | One-way ANOVA + Tukey |
|  | *Prg2* | 0.5245 / 0.8644 / 0.8134 / 0.8386 / 0.9529 | 0.8041 | One-way ANOVA + Tukey |
| Figure 2C | IFN-γ | 0.4040 / 0.2231 / 0.1618 / 0.4891 / 0.6453 | 0.3023 | One-way ANOVA + Tukey |
|  | PRG2 | 0.4249 / 0.6969 / 0.0011 / 0.8562 / 0.1463 | 0.4342 | Kruskal–Wallis + Dunn |
| Figure 2D | *Gata3* | 0.3112 / 0.2450 / 0.2657 / 0.8290 / 0.8388 | 0.6822 | One-way ANOVA + Tukey |
|  | *Il4* | 0.1222 / 0.5971 / 0.2042 / 0.1382 / 0.5826 | 0.0604 | One-way ANOVA + Tukey |
| Figure 2E | *Foxp3* | 0.4771 / 0.3978 / 0.4156 / 0.6217 / 0.0024 | 0.0147 | Kruskal–Wallis + Dunn |
|  | *Il10* | 0.1388 / 0.0268 / 0.7890 / 0.1871 / 0.2299 | 0.0366 | Kruskal–Wallis + Dunn |
| Figure 2F | IL-4 | 0.6691 / 0.9746 / 0.3230 / 0.9028 / 0.7823 | 0.5064 | One-way ANOVA + Tukey |
|  | IL-10 | 0.4054 / 0.1009 / 0.6347 / 0.7317 / 0.4399 | 0.2771 | One-way ANOVA + Tukey |
| Figure 3B | TLR4/β-actin | 0.6149 / 0.1894 / 0.6193 / 0.8361 / 0.6785 | 0.0753 | One-way ANOVA + Tukey |
| Figure 3C | p-p65/p65 | 0.7026 / 0.2257 / 0.7442 / 0.8483 / 0.5948 | 0.0463 | Welch’s one-way ANOVA + Games–Howell |
| Figure 3D | p-IκB/β-actin | 0.3615 / 0.1245 / 0.8785 / 0.2087 / 0.0167 | 0.0866 | Kruskal–Wallis + Dunn |

**Note:** Shapiro–Wilk *P* values are presented in the following group order: Ctrl, Pp-14d, Pp-28d, Pp-42d, and Pp-TCBZ. If any group failed the normality test, the Kruskal–Wallis test followed by Dunn’s multiple-comparisons test was used. When all groups satisfied the normality assumption, ordinary one-way ANOVA followed by Tukey’s test was used if Levene’s test indicated homogeneity of variance; otherwise, Welch’s one-way ANOVA followed by the Games–Howell test was used. For qRT-PCR outcomes, assumption testing and statistical analyses were performed using ΔCt values.

**Table F. Omnibus statistical results, effect sizes, and Benjamini–Hochberg FDR correction by outcome family**

| **Figure panel** | **Outcome** | **Global test** | **Raw *P*** | **FDR-adjusted *P*** | **Effect size** | **95% CI** |
| --- | --- | --- | --- | --- | --- | --- |
| Figure 1F | Relative ATS lung injury score | One-way ANOVA, *F*(4, 20) = 1135.817 | 2.87 × 10⁻²³ | 5.73 × 10⁻²³ | η² = 0.996 | 0.995–0.998 |
| Figure 1G | Collagen area fraction (CAF) | One-way ANOVA, *F*(4, 20) = 257.685 | 6.75 × 10⁻¹⁷ | 6.75 × 10⁻¹⁷ | η² = 0.981 | 0.975–0.994 |
| Figure 1H | *Il1b* | Welch’s one-way ANOVA, *F*(4, 9.85) = 12.135 | 0.000795 | 0.000851 | η² = 0.668 | 0.560–0.901 |
|  | *Tnfa* | One-way ANOVA, *F*(4, 20) = 11.791 | 4.40 × 10⁻⁵ | 0.000110 | η² = 0.702 | 0.595–0.897 |
| Figure 2A | *Tbx21* | One-way ANOVA, *F*(4, 20) = 33.297 | 1.40 × 10⁻⁸ | 6.98 × 10⁻⁸ | η² = 0.869 | 0.833–0.959 |
|  | *Ifng* | Kruskal–Wallis, *H*(4) = 18.823 | 0.000851 | 0.000851 | ε² = 0.741 | 0.729–0.893 |
| Figure 2B | *Epx* | One-way ANOVA, *F*(4, 20) = 10.791 | 7.94 × 10⁻⁵ | 0.000132 | η² = 0.683 | 0.587–0.877 |
|  | *Prg2* | One-way ANOVA, *F*(4, 20) = 36.304 | 6.61 × 10⁻⁹ | 6.61 × 10⁻⁸ | η² = 0.879 | 0.845–0.951 |
| Figure 2C | IFN-γ | One-way ANOVA, *F*(4, 20) = 13.632 | 1.61 × 10⁻⁵ | 3.22 × 10⁻⁵ | η² = 0.732 | 0.600–0.928 |
|  | PRG2 | Kruskal–Wallis, *H*(4) = 20.671 | 0.000368 | 0.000368 | ε² = 0.834 | 0.756–0.936 |
| Figure 2D | *Gata3* | One-way ANOVA, *F*(4, 20) = 10.940 | 7.25 × 10⁻⁵ | 0.000132 | η² = 0.686 | 0.604–0.868 |
|  | *Il4* | One-way ANOVA, *F*(4, 20) = 26.171 | 1.06 × 10⁻⁷ | 3.53 × 10⁻⁷ | η² = 0.840 | 0.793–0.963 |
| Figure 2E | *Foxp3* | Kruskal–Wallis, *H*(4) = 21.319 | 0.000274 | 0.000391 | ε² = 0.866 | 0.846–0.932 |
|  | *Il10* | Kruskal–Wallis, *H*(4) = 20.788 | 0.000349 | 0.000436 | ε² = 0.839 | 0.805–0.926 |
| Figure 2F | IL-4 | One-way ANOVA, *F*(4, 20) = 12.833 | 2.46 × 10⁻⁵ | 3.28 × 10⁻⁵ | η² = 0.720 | 0.654–0.886 |
|  | IL-10 | One-way ANOVA, *F*(4, 20) = 33.033 | 1.49 × 10⁻⁸ | 5.97 × 10⁻⁸ | η² = 0.869 | 0.834–0.959 |
| Figure 3B | TLR4/β-actin | One-way ANOVA, *F*(4, 10) = 3.984 | 0.034687 | 0.034687 | η² = 0.614 | 0.548–0.952 |
| Figure 3C | p-p65/p65 | Welch’s one-way ANOVA, *F*(4, 4.77) = 77.187 | 0.000154 | 0.000463 | η² = 0.804 | 0.740–0.986 |
| Figure 3D | p-IκB/β-actin | Kruskal–Wallis, *H*(4) = 10.667 | 0.030577 | 0.034687 | ε² = 0.667 | 0.640–0.927 |
| Figure 4A | Sobs | Kruskal–Wallis, *H*(4) = 16.800 | 0.002114 | 0.002818 | ε² = 0.512 | NE |
|  | Chao1 | Kruskal–Wallis, *H*(4) = 16.800 | 0.002114 | 0.002818 | ε² = 0.512 | NE |
|  | Shannon | Kruskal–Wallis, *H*(4) = 17.398 | 0.001617 | 0.002818 | ε² = 0.536 | NE |
|  | Simpson | Kruskal–Wallis, *H*(4) = 14.353 | 0.006250 | 0.006250 | ε² = 0.414 | NE |
| Figure 4B | Bray–Curtis community composition | ANOSIM | 0.001000 | NA | *R* = 0.3061 | NE |

**Note:** Benjamini–Hochberg FDR correction was performed separately within the outcome families defined in Table D. For qRT-PCR outcomes, statistical analyses were performed using ΔCt values. For the two prespecified primary outcomes, post hoc achieved power was >0.999 for both the relative ATS lung injury score and collagen area fraction (CAF), based on a fixed-effects omnibus one-way ANOVA with five groups, five animals per group, and *α* = 0.05. Cohen’s *f* was calculated as √[η²/(1 − η²)] and was 15.072 for the relative ATS lung injury score and 7.179 for CAF. η², eta squared; ε², epsilon squared; ANOSIM, analysis of similarities; NA, not applicable; NE, not estimable.

**Table G. Post hoc pairwise comparisons and multiplicity-adjusted *P* values**

| **Figure panel** | **Outcome** | **Comparison** | **Post hoc test** | **Raw *P*** | **Post hoc-adjusted *P*** | **Pairwise effect size** | **95% CI** |
| --- | --- | --- | --- | --- | --- | --- | --- |
| Figure 1F | Relative ATS lung injury score | Ctrl vs Pp-14d | Tukey | 1.27 × 10⁻⁵ | 0.000112 | Hedges’ g = −4.228 | −8.274 to −3.415 |
|  | Relative ATS lung injury score | Ctrl vs Pp-28d | Tukey | 1.38 × 10⁻¹⁶ | 2.11 × 10⁻¹⁴ | Hedges’ g = −14.623 | −28.392 to −12.290 |
|  | Relative ATS lung injury score | Ctrl vs Pp-42d | Tukey | 3.65 × 10⁻²⁴ | 1.92 × 10⁻¹⁴ | Hedges’ g = −36.570 | −69.980 to −30.955 |
|  | Relative ATS lung injury score | Ctrl vs Pp-TCBZ | Tukey | 2.69 × 10⁻¹⁷ | 1.95 × 10⁻¹⁴ | Hedges’ g = −16.778 | −32.476 to −14.238 |
|  | Relative ATS lung injury score | Pp-14d vs Pp-28d | Tukey | 2.06 × 10⁻¹⁴ | 1.91 × 10⁻¹³ | Hedges’ g = −10.979 | −21.189 to −9.174 |
|  | Relative ATS lung injury score | Pp-14d vs Pp-42d | Tukey | 2.63 × 10⁻²³ | 1.92 × 10⁻¹⁴ | Hedges’ g = −32.194 | −61.592 to −27.300 |
|  | Relative ATS lung injury score | Pp-14d vs Pp-TCBZ | Tukey | 2.62 × 10⁻¹⁵ | 4.31 × 10⁻¹⁴ | Hedges’ g = −12.862 | −24.854 to −10.831 |
|  | Relative ATS lung injury score | Pp-28d vs Pp-42d | Tukey | 1.40 × 10⁻¹⁹ | 1.92 × 10⁻¹⁴ | Hedges’ g = −17.805 | −32.714 to −15.307 |
|  | Relative ATS lung injury score | Pp-28d vs Pp-TCBZ | Tukey | 0.039594 | 0.219394 | Hedges’ g = −1.115 | −3.081 to −0.132 |
|  | Relative ATS lung injury score | Pp-42d vs Pp-TCBZ | Tukey | 4.89 × 10⁻¹⁹ | 1.92 × 10⁻¹⁴ | Hedges’ g = 17.361 | 14.882 to 32.715 |
| Figure 1G | Collagen area fraction (CAF) | Ctrl vs Pp-14d | Tukey | 9.61 × 10⁻⁵ | 0.000818 | Hedges’ g = −4.457 | −9.068 to −3.962 |
|  | Collagen area fraction (CAF) | Ctrl vs Pp-28d | Tukey | 1.16 × 10⁻¹¹ | 1.10 × 10⁻¹⁰ | Hedges’ g = −6.755 | −18.412 to −5.135 |
| Figure 1G | Collagen area fraction (CAF) | Ctrl vs Pp-42d | Tukey | 7.33 × 10⁻¹⁸ | 1.93 × 10⁻¹⁴ | Hedges’ g = −16.219 | −34.224 to −12.894 |
|  | Collagen area fraction (CAF) | Ctrl vs Pp-TCBZ | Tukey | 1.99 × 10⁻⁶ | 1.80 × 10⁻⁵ | Hedges’ g = −5.834 | −13.003 to −4.953 |
|  | Collagen area fraction (CAF) | Pp-14d vs Pp-28d | Tukey | 2.12 × 10⁻⁸ | 1.97 × 10⁻⁷ | Hedges’ g = −4.524 | −14.769 to −3.205 |
|  | Collagen area fraction (CAF) | Pp-14d vs Pp-42d | Tukey | 2.54 × 10⁻¹⁶ | 2.24 × 10⁻¹⁴ | Hedges’ g = −14.129 | −33.461 to −10.903 |
|  | Collagen area fraction (CAF) | Pp-14d vs Pp-TCBZ | Tukey | 0.096653 | 0.431826 | Hedges’ g = −1.738 | −5.607 to −0.684 |
|  | Collagen area fraction (CAF) | Pp-28d vs Pp-42d | Tukey | 1.50 × 10⁻¹² | 1.46 × 10⁻¹¹ | Hedges’ g = −6.322 | −13.107 to −5.240 |
|  | Collagen area fraction (CAF) | Pp-28d vs Pp-TCBZ | Tukey | 6.08 × 10⁻⁷ | 5.55 × 10⁻⁶ | Hedges’ g = 3.596 | 2.483 to 11.122 |
|  | Collagen area fraction (CAF) | Pp-42d vs Pp-TCBZ | Tukey | 1.07 × 10⁻¹⁵ | 3.03 × 10⁻¹⁴ | Hedges’ g = 12.915 | 10.116 to 28.846 |
| Figure 1H | *Il1b* | Ctrl vs Pp-14d | Games–Howell | 0.013653 | 0.065063 | Hedges’ g = 2.152 | 1.483 to 5.784 |
|  | *Il1b* | Ctrl vs Pp-28d | Games–Howell | 0.014348 | 0.067450 | Hedges’ g = 2.152 | 1.499 to 5.788 |
|  | *Il1b* | Ctrl vs Pp-42d | Games–Howell | 0.180076 | 0.569035 | Hedges’ g = 0.899 | −0.157 to 3.444 |
|  | *Il1b* | Ctrl vs Pp-TCBZ | Games–Howell | 0.204952 | 0.628304 | Hedges’ g = 0.826 | −0.241 to 3.061 |
|  | *Il1b* | Pp-14d vs Pp-28d | Games–Howell | 0.948384 | 0.999994 | Hedges’ g = −0.038 | −1.493 to 1.219 |
| Figure 1H | *Il1b* | Pp-14d vs Pp-42d | Games–Howell | 0.000852 | 0.005515 | Hedges’ g = −2.971 | −5.598 to −2.385 |
|  | *Il1b* | Pp-14d vs Pp-TCBZ | Games–Howell | 0.001894 | 0.011818 | Hedges’ g = −2.634 | −5.348 to −1.998 |
|  | *Il1b* | Pp-28d vs Pp-42d | Games–Howell | 0.000681 | 0.004451 | Hedges’ g = −3.061 | −5.413 to −2.671 |
|  | *Il1b* | Pp-28d vs Pp-TCBZ | Games–Howell | 0.001855 | 0.011459 | Hedges’ g = −2.683 | −5.336 to −2.172 |
|  | *Il1b* | Pp-42d vs Pp-TCBZ | Games–Howell | 0.873393 | 0.999791 | Hedges’ g = −0.094 | −1.534 to 1.252 |
|  | *Tnfa* | Ctrl vs Pp-14d | Tukey | 0.000319 | 0.002628 | Hedges’ g = 1.897 | 1.267 to 7.064 |
|  | *Tnfa* | Ctrl vs Pp-28d | Tukey | 4.77 × 10⁻⁵ | 0.000413 | Hedges’ g = 2.062 | 1.410 to 4.935 |
|  | *Tnfa* | Ctrl vs Pp-42d | Tukey | 3.25 × 10⁻⁶ | 2.92 × 10⁻⁵ | Hedges’ g = 2.769 | 1.991 to 10.849 |
|  | *Tnfa* | Ctrl vs Pp-TCBZ | Tukey | 0.004869 | 0.034951 | Hedges’ g = 1.355 | 0.637 to 4.194 |
|  | *Tnfa* | Pp-14d vs Pp-28d | Tukey | 0.421628 | 0.921195 | Hedges’ g = 0.616 | −0.636 to 2.163 |
|  | *Tnfa* | Pp-14d vs Pp-42d | Tukey | 0.055775 | 0.287837 | Hedges’ g = 2.265 | 1.282 to 6.259 |
|  | *Tnfa* | Pp-14d vs Pp-TCBZ | Tukey | 0.254412 | 0.765927 | Hedges’ g = −1.192 | −2.989 to −0.260 |
| Figure 1H | *Tnfa* | Pp-28d vs Pp-42d | Tukey | 0.240233 | 0.745565 | Hedges’ g = 0.894 | −0.125 to 4.578 |
|  | *Tnfa* | Pp-28d vs Pp-TCBZ | Tukey | 0.059981 | 0.304401 | Hedges’ g = −1.410 | −3.236 to −0.636 |
|  | *Tnfa* | Pp-42d vs Pp-TCBZ | Tukey | 0.004452 | 0.032180 | Hedges’ g = −3.164 | −6.890 to −2.260 |
| Figure 2A | *Tbx21* | Ctrl vs Pp-14d | Tukey | 4.05 × 10⁻⁹ | 4.18 × 10⁻⁸ | Hedges’ g = 4.227 | 3.383 to 12.870 |
|  | *Tbx21* | Ctrl vs Pp-28d | Tukey | 1.14 × 10⁻⁸ | 1.10 × 10⁻⁷ | Hedges’ g = 3.847 | 3.132 to 9.807 |
|  | *Tbx21* | Ctrl vs Pp-42d | Tukey | 5.74 × 10⁻⁸ | 5.35 × 10⁻⁷ | Hedges’ g = 3.669 | 2.910 to 12.695 |
|  | *Tbx21* | Ctrl vs Pp-TCBZ | Tukey | 5.64 × 10⁻⁵ | 0.000486 | Hedges’ g = 2.190 | 1.675 to 6.179 |
|  | *Tbx21* | Pp-14d vs Pp-28d | Tukey | 0.556220 | 0.973704 | Hedges’ g = −0.448 | −2.533 to 0.647 |
|  | *Tbx21* | Pp-14d vs Pp-42d | Tukey | 0.153840 | 0.584867 | Hedges’ g = −1.354 | −3.577 to −0.453 |
|  | *Tbx21* | Pp-14d vs Pp-TCBZ | Tukey | 0.000117 | 0.000995 | Hedges’ g = −4.049 | −11.084 to −2.845 |
|  | *Tbx21* | Pp-28d vs Pp-42d | Tukey | 0.387296 | 0.899531 | Hedges’ g = −0.709 | −2.023 to 0.418 |
|  | *Tbx21* | Pp-28d vs Pp-TCBZ | Tukey | 0.000473 | 0.003848 | Hedges’ g = −3.160 | −7.206 to −2.297 |
|  | *Tbx21* | Pp-42d vs Pp-TCBZ | Tukey | 0.003695 | 0.027073 | Hedges’ g = −3.054 | −10.672 to −1.785 |
| Figure 2A | *Ifng* | Ctrl vs Pp-14d | Dunn | 0.000426 | 0.004262 | Cliff’s δ = 1.000 | 1.000 to 1.000 |
|  | *Ifng* | Ctrl vs Pp-28d | Dunn | 0.001271 | 0.012707 | Cliff’s δ = 1.000 | 1.000 to 1.000 |
|  | *Ifng* | Ctrl vs Pp-42d | Dunn | 0.003481 | 0.034807 | Cliff’s δ = 1.000 | 1.000 to 1.000 |
|  | *Ifng* | Ctrl vs Pp-TCBZ | Dunn | 0.282745 | 1.000000 | Cliff’s δ = 1.000 | 1.000 to 1.000 |
|  | *Ifng* | Pp-14d vs Pp-28d | Dunn | 0.763591 | 1.000000 | Cliff’s δ = −0.120 | −0.920 to 0.680 |
|  | *Ifng* | Pp-14d vs Pp-42d | Dunn | 0.547483 | 1.000000 | Cliff’s δ = −0.440 | −1.000 to 0.280 |
|  | *Ifng* | Pp-14d vs Pp-TCBZ | Dunn | 0.014321 | 0.143209 | Cliff’s δ = −1.000 | −1.000 to −1.000 |
|  | *Ifng* | Pp-28d vs Pp-42d | Dunn | 0.763591 | 1.000000 | Cliff’s δ = −0.120 | −0.840 to 0.680 |
|  | *Ifng* | Pp-28d vs Pp-TCBZ | Dunn | 0.031686 | 0.316864 | Cliff’s δ = −1.000 | −1.000 to −1.000 |
|  | *Ifng* | Pp-42d vs Pp-TCBZ | Dunn | 0.064664 | 0.646636 | Cliff’s δ = −1.000 | −1.000 to −1.000 |
| Figure 2B | *Epx* | Ctrl vs Pp-14d | Tukey | 0.000454 | 0.003700 | Hedges’ g = 2.249 | 1.446 to 5.588 |
|  | *Epx* | Ctrl vs Pp-28d | Tukey | 2.32 × 10⁻⁵ | 0.000203 | Hedges’ g = 2.593 | 1.702 to 5.881 |
| Figure 2B | *Epx* | Ctrl vs Pp-42d | Tukey | 1.85 × 10⁻⁵ | 0.000163 | Hedges’ g = 3.664 | 3.140 to 11.584 |
|  | *Epx* | Ctrl vs Pp-TCBZ | Tukey | 0.014427 | 0.093187 | Hedges’ g = 1.335 | 0.594 to 2.920 |
|  | *Epx* | Pp-14d vs Pp-28d | Tukey | 0.211857 | 0.700227 | Hedges’ g = 0.687 | −0.402 to 2.805 |
|  | *Epx* | Pp-14d vs Pp-42d | Tukey | 0.179755 | 0.640572 | Hedges’ g = 1.185 | 0.517 to 3.196 |
|  | *Epx* | Pp-14d vs Pp-TCBZ | Tukey | 0.147200 | 0.569369 | Hedges’ g = −0.859 | −2.943 to 0.197 |
|  | *Epx* | Pp-28d vs Pp-42d | Tukey | 0.920999 | 0.999975 | Hedges’ g = 0.065 | −2.129 to 1.248 |
|  | *Epx* | Pp-28d vs Pp-TCBZ | Tukey | 0.011114 | 0.073950 | Hedges’ g = −1.385 | −4.800 to −0.356 |
|  | *Epx* | Pp-42d vs Pp-TCBZ | Tukey | 0.008891 | 0.060537 | Hedges’ g = −2.082 | −12.847 to −1.347 |
|  | *Prg2* | Ctrl vs Pp-14d | Tukey | 2.45 × 10⁻⁷ | 2.25 × 10⁻⁶ | Hedges’ g = 4.701 | 3.683 to 9.934 |
|  | *Prg2* | Ctrl vs Pp-28d | Tukey | 1.23 × 10⁻⁹ | 1.55 × 10⁻⁸ | Hedges’ g = 6.747 | 5.471 to 13.649 |
|  | *Prg2* | Ctrl vs Pp-42d | Tukey | 1.40 × 10⁻⁸ | 1.34 × 10⁻⁷ | Hedges’ g = 5.077 | 4.068 to 10.438 |
|  | *Prg2* | Ctrl vs Pp-TCBZ | Tukey | 0.000556 | 0.004494 | Hedges’ g = 2.001 | 1.186 to 4.615 |
|  | *Prg2* | Pp-14d vs Pp-28d | Tukey | 0.007868 | 0.054202 | Hedges’ g = 2.177 | 1.308 to 4.867 |
| Figure 2B | *Prg2* | Pp-14d vs Pp-42d | Tukey | 0.143902 | 0.561473 | Hedges’ g = 0.939 | −0.024 to 2.577 |
|  | *Prg2* | Pp-14d vs Pp-TCBZ | Tukey | 0.002153 | 0.016330 | Hedges’ g = −1.859 | −4.698 to −0.974 |
|  | *Prg2* | Pp-28d vs Pp-42d | Tukey | 0.167659 | 0.615479 | Hedges’ g = −0.914 | −3.121 to 0.123 |
|  | *Prg2* | Pp-28d vs Pp-TCBZ | Tukey | 2.60 × 10⁻⁶ | 2.34 × 10⁻⁵ | Hedges’ g = −3.504 | −7.941 to −2.521 |
|  | *Prg2* | Pp-42d vs Pp-TCBZ | Tukey | 6.25 × 10⁻⁵ | 0.000538 | Hedges’ g = −2.460 | −5.531 to −1.617 |
| Figure 2C | IFN-γ | Ctrl vs Pp-14d | Tukey | 8.69 × 10⁻⁶ | 7.72 × 10⁻⁵ | Hedges’ g = −2.830 | −9.670 to −1.657 |
|  | IFN-γ | Ctrl vs Pp-28d | Tukey | 0.000116 | 0.000982 | Hedges’ g = −3.372 | −11.410 to −2.140 |
|  | IFN-γ | Ctrl vs Pp-42d | Tukey | 0.001894 | 0.014474 | Hedges’ g = −2.880 | −6.905 to −2.205 |
|  | IFN-γ | Ctrl vs Pp-TCBZ | Tukey | 0.627889 | 0.987193 | Hedges’ g = −0.374 | −1.811 to 0.881 |
|  | IFN-γ | Pp-14d vs Pp-28d | Tukey | 0.266271 | 0.781880 | Hedges’ g = 0.509 | −0.599 to 3.060 |
|  | IFN-γ | Pp-14d vs Pp-42d | Tukey | 0.029641 | 0.172918 | Hedges’ g = 1.093 | 0.040 to 5.780 |
|  | IFN-γ | Pp-14d vs Pp-TCBZ | Tukey | 2.61 × 10⁻⁵ | 0.000228 | Hedges’ g = 2.482 | 1.373 to 8.024 |
| Figure 2C | IFN-γ | Pp-28d vs Pp-42d | Tukey | 0.244788 | 0.752264 | Hedges’ g = 0.804 | −0.225 to 4.591 |
|  | IFN-γ | Pp-28d vs Pp-TCBZ | Tukey | 0.000364 | 0.002991 | Hedges’ g = 2.758 | 1.614 to 9.383 |
|  | IFN-γ | Pp-42d vs Pp-TCBZ | Tukey | 0.005868 | 0.041491 | Hedges’ g = 2.210 | 1.468 to 5.541 |
|  | PRG2 | Ctrl vs Pp-14d | Dunn | 0.047972 | 0.479718 | Cliff’s δ = −0.920 | −1.000 to −0.520 |
|  | PRG2 | Ctrl vs Pp-28d | Dunn | 3.67 × 10⁻⁵ | 0.000367 | Cliff’s δ = −1.000 | −1.000 to −1.000 |
|  | PRG2 | Ctrl vs Pp-42d | Dunn | 0.004548 | 0.045475 | Cliff’s δ = −1.000 | −1.000 to −1.000 |
|  | PRG2 | Ctrl vs Pp-TCBZ | Dunn | 0.344242 | 1.000000 | Cliff’s δ = −0.760 | −1.000 to −0.200 |
|  | PRG2 | Pp-14d vs Pp-28d | Dunn | 0.031588 | 0.315880 | Cliff’s δ = −1.000 | −1.000 to −1.000 |
|  | PRG2 | Pp-14d vs Pp-42d | Dunn | 0.389881 | 1.000000 | Cliff’s δ = −0.600 | −1.000 to 0.120 |
|  | PRG2 | Pp-14d vs Pp-TCBZ | Dunn | 0.302165 | 1.000000 | Cliff’s δ = 0.680 | 0.040 to 1.000 |
|  | PRG2 | Pp-28d vs Pp-42d | Dunn | 0.197137 | 1.000000 | Cliff’s δ = 1.000 | 1.000 to 1.000 |
|  | PRG2 | Pp-28d vs Pp-TCBZ | Dunn | 0.001466 | 0.014657 | Cliff’s δ = 1.000 | 1.000 to 1.000 |
|  | PRG2 | Pp-42d vs Pp-TCBZ | Dunn | 0.058540 | 0.585396 | Cliff’s δ = 1.000 | 1.000 to 1.000 |
| Figure 2D | *Gata3* | Ctrl vs Pp-14d | Tukey | 0.000145 | 0.001224 | Hedges’ g = 2.602 | 1.937 to 5.781 |
|  | *Gata3* | Ctrl vs Pp-28d | Tukey | 0.000134 | 0.001130 | Hedges’ g = 2.444 | 1.866 to 5.952 |
|  | *Gata3* | Ctrl vs Pp-42d | Tukey | 1.30 × 10⁻⁵ | 0.000115 | Hedges’ g = 2.972 | 2.240 to 6.529 |
|  | *Gata3* | Ctrl vs Pp-TCBZ | Tukey | 0.043040 | 0.234652 | Hedges’ g = 1.318 | 0.396 to 4.350 |
|  | *Gata3* | Pp-14d vs Pp-28d | Tukey | 0.972218 | 1.000000 | Hedges’ g = 0.020 | −1.462 to 1.241 |
|  | *Gata3* | Pp-14d vs Pp-42d | Tukey | 0.302569 | 0.825179 | Hedges’ g = 0.596 | −0.572 to 2.227 |
|  | *Gata3* | Pp-14d vs Pp-TCBZ | Tukey | 0.020530 | 0.126669 | Hedges’ g = −1.729 | −3.830 to −0.993 |
|  | *Gata3* | Pp-28d vs Pp-42d | Tukey | 0.318544 | 0.841836 | Hedges’ g = 0.536 | −0.552 to 2.495 |
|  | *Gata3* | Pp-28d vs Pp-TCBZ | Tukey | 0.019035 | 0.118673 | Hedges’ g = −1.580 | −3.320 to −0.971 |
|  | *Gata3* | Pp-42d vs Pp-TCBZ | Tukey | 0.001900 | 0.014512 | Hedges’ g = −2.211 | −4.767 to −1.448 |
|  | *Il4* | Ctrl vs Pp-14d | Tukey | 3.14 × 10⁻⁵ | 0.000273 | Hedges’ g = 2.084 | 1.662 to 6.027 |
|  | *Il4* | Ctrl vs Pp-28d | Tukey | 3.71 × 10⁻⁸ | 3.48 × 10⁻⁷ | Hedges’ g = 3.313 | 2.734 to 9.622 |
| Figure 2D | *Il4* | Ctrl vs Pp-42d | Tukey | 1.75 × 10⁻⁸ | 1.66 × 10⁻⁷ | Hedges’ g = 3.463 | 2.877 to 10.041 |
|  | *Il4* | Ctrl vs Pp-TCBZ | Tukey | 4.87 × 10⁻⁵ | 0.000420 | Hedges’ g = 2.007 | 1.604 to 5.739 |
|  | *Il4* | Pp-14d vs Pp-28d | Tukey | 0.003904 | 0.028492 | Hedges’ g = 4.091 | 3.607 to 8.163 |
|  | *Il4* | Pp-14d vs Pp-42d | Tukey | 0.001503 | 0.011628 | Hedges’ g = 4.490 | 3.890 to 10.829 |
|  | *Il4* | Pp-14d vs Pp-TCBZ | Tukey | 0.848883 | 0.999661 | Hedges’ g = −0.280 | −2.557 to 0.804 |
|  | *Il4* | Pp-28d vs Pp-42d | Tukey | 0.684046 | 0.993406 | Hedges’ g = 0.450 | −0.801 to 2.030 |
|  | *Il4* | Pp-28d vs Pp-TCBZ | Tukey | 0.002504 | 0.018820 | Hedges’ g = −4.285 | −9.572 to −3.438 |
|  | *Il4* | Pp-42d vs Pp-TCBZ | Tukey | 0.000958 | 0.007576 | Hedges’ g = −4.676 | −13.706 to −3.783 |
| Figure 2E | *Foxp3* | Ctrl vs Pp-14d | Dunn | 0.212750 | 1.000000 | Cliff’s δ = 1.000 | 1.000 to 1.000 |
|  | *Foxp3* | Ctrl vs Pp-28d | Dunn | 0.000220 | 0.002198 | Cliff’s δ = 1.000 | 1.000 to 1.000 |
|  | *Foxp3* | Ctrl vs Pp-42d | Dunn | 0.000131 | 0.001313 | Cliff’s δ = 1.000 | 1.000 to 1.000 |
|  | *Foxp3* | Ctrl vs Pp-TCBZ | Dunn | 0.048101 | 0.481008 | Cliff’s δ = 1.000 | 1.000 to 1.000 |
|  | *Foxp3* | Pp-14d vs Pp-28d | Dunn | 0.014321 | 0.143209 | Cliff’s δ = 1.000 | 1.000 to 1.000 |
| Figure 2E | *Foxp3* | Pp-14d vs Pp-42d | Dunn | 0.009937 | 0.099370 | Cliff’s δ = 1.000 | 1.000 to 1.000 |
|  | *Foxp3* | Pp-14d vs Pp-TCBZ | Dunn | 0.465123 | 1.000000 | Cliff’s δ = 0.680 | 0.040 to 1.000 |
|  | *Foxp3* | Pp-28d vs Pp-42d | Dunn | 0.897436 | 1.000000 | Cliff’s δ = 0.120 | −0.680 to 0.840 |
|  | *Foxp3* | Pp-28d vs Pp-TCBZ | Dunn | 0.085673 | 0.856734 | Cliff’s δ = −1.000 | −1.000 to −1.000 |
|  | *Foxp3* | Pp-42d vs Pp-TCBZ | Dunn | 0.064664 | 0.646636 | Cliff’s δ = −1.000 | −1.000 to −1.000 |
|  | *Il10* | Ctrl vs Pp-14d | Dunn | 0.064664 | 0.646636 | Cliff’s δ = 1.000 | 1.000 to 1.000 |
|  | *Il10* | Ctrl vs Pp-28d | Dunn | 0.001977 | 0.019773 | Cliff’s δ = 1.000 | 1.000 to 1.000 |
|  | *Il10* | Ctrl vs Pp-42d | Dunn | 2.10 × 10⁻⁵ | 0.000210 | Cliff’s δ = 1.000 | 1.000 to 1.000 |
|  | *Il10* | Ctrl vs Pp-TCBZ | Dunn | 0.121910 | 1.000000 | Cliff’s δ = 1.000 | 1.000 to 1.000 |
|  | *Il10* | Pp-14d vs Pp-28d | Dunn | 0.212750 | 1.000000 | Cliff’s δ = 0.680 | 0.040 to 1.000 |
|  | *Il10* | Pp-14d vs Pp-42d | Dunn | 0.016122 | 0.161218 | Cliff’s δ = 1.000 | 1.000 to 1.000 |
|  | *Il10* | Pp-14d vs Pp-TCBZ | Dunn | 0.763591 | 1.000000 | Cliff’s δ = −0.120 | −0.840 to 0.680 |
| Figure 2E | *Il10* | Pp-28d vs Pp-42d | Dunn | 0.246006 | 1.000000 | Cliff’s δ = 0.920 | 0.520 to 1.000 |
|  | *Il10* | Pp-28d vs Pp-TCBZ | Dunn | 0.121910 | 1.000000 | Cliff’s δ = −1.000 | −1.000 to −1.000 |
|  | *Il10* | Pp-42d vs Pp-TCBZ | Dunn | 0.006791 | 0.067912 | Cliff’s δ = −1.000 | −1.000 to −1.000 |
| Figure 2F | IL-4 | Ctrl vs Pp-14d | Tukey | 0.007119 | 0.049495 | Hedges’ g = −2.680 | −5.786 to −1.910 |
|  | IL-4 | Ctrl vs Pp-28d | Tukey | 0.000185 | 0.001551 | Hedges’ g = −2.619 | −7.795 to −1.952 |
|  | IL-4 | Ctrl vs Pp-42d | Tukey | 1.16 × 10⁻⁶ | 1.05 × 10⁻⁵ | Hedges’ g = −5.161 | −10.087 to −4.129 |
|  | IL-4 | Ctrl vs Pp-TCBZ | Tukey | 0.016843 | 0.106718 | Hedges’ g = −1.528 | −3.745 to −0.844 |
|  | IL-4 | Pp-14d vs Pp-28d | Tukey | 0.131145 | 0.529624 | Hedges’ g = −0.851 | −2.297 to 0.152 |
|  | IL-4 | Pp-14d vs Pp-42d | Tukey | 0.000978 | 0.007727 | Hedges’ g = −2.639 | −5.460 to −1.889 |
|  | IL-4 | Pp-14d vs Pp-TCBZ | Tukey | 0.700944 | 0.994721 | Hedges’ g = 0.215 | −1.024 to 2.069 |
|  | IL-4 | Pp-28d vs Pp-42d | Tukey | 0.033388 | 0.190862 | Hedges’ g = −1.152 | −3.789 to −0.092 |
|  | IL-4 | Pp-28d vs Pp-TCBZ | Tukey | 0.063615 | 0.318351 | Hedges’ g = 0.871 | −0.142 to 2.609 |
|  | IL-4 | Pp-42d vs Pp-TCBZ | Tukey | 0.000394 | 0.003221 | Hedges’ g = 2.179 | 1.345 to 5.407 |
| Figure 2F | IL-10 | Ctrl vs Pp-14d | Tukey | 1.78 × 10⁻⁵ | 0.000156 | Hedges’ g = −2.865 | −11.802 to −1.543 |
|  | IL-10 | Ctrl vs Pp-28d | Tukey | 6.27 × 10⁻⁹ | 6.25 × 10⁻⁸ | Hedges’ g = −5.884 | −12.973 to −4.947 |
|  | IL-10 | Ctrl vs Pp-42d | Tukey | 2.73 × 10⁻⁹ | 2.94 × 10⁻⁸ | Hedges’ g = −7.621 | −17.180 to −6.119 |
|  | IL-10 | Ctrl vs Pp-TCBZ | Tukey | 2.62 × 10⁻⁵ | 0.000229 | Hedges’ g = −5.730 | −11.051 to −4.789 |
|  | IL-10 | Pp-14d vs Pp-28d | Tukey | 0.000698 | 0.005593 | Hedges’ g = −1.707 | −3.478 to −1.256 |
|  | IL-10 | Pp-14d vs Pp-42d | Tukey | 0.000222 | 0.001850 | Hedges’ g = −2.097 | −5.845 to −1.567 |
|  | IL-10 | Pp-14d vs Pp-TCBZ | Tukey | 0.864454 | 0.999781 | Hedges’ g = 0.089 | −0.911 to 2.584 |
|  | IL-10 | Pp-28d vs Pp-42d | Tukey | 0.629327 | 0.987393 | Hedges’ g = −0.264 | −2.228 to 0.892 |
|  | IL-10 | Pp-28d vs Pp-TCBZ | Tukey | 0.000466 | 0.003790 | Hedges’ g = 2.599 | 2.000 to 5.202 |
|  | IL-10 | Pp-42d vs Pp-TCBZ | Tukey | 0.000149 | 0.001251 | Hedges’ g = 3.608 | 2.631 to 8.990 |
| Figure 3B | TLR4/β-actin | Ctrl vs Pp-14d | Tukey | 0.112890 | 0.455359 | Hedges’ g = −2.277 | −23.350 to −1.460 |
|  | TLR4/β-actin | Ctrl vs Pp-28d | Tukey | 0.003707 | 0.024072 | Hedges’ g = −8.478 | −30.484 to −7.646 |
| Figure 3B | TLR4/β-actin | Ctrl vs Pp-42d | Tukey | 0.070627 | 0.321976 | Hedges’ g = −1.805 | −13.269 to −1.066 |
|  | TLR4/β-actin | Ctrl vs Pp-TCBZ | Tukey | 0.415243 | 0.908542 | Hedges’ g = −0.418 | −9.010 to 4.656 |
|  | TLR4/β-actin | Pp-14d vs Pp-28d | Tukey | 0.070424 | 0.321263 | Hedges’ g = −2.671 | −16.396 to −2.350 |
|  | TLR4/β-actin | Pp-14d vs Pp-42d | Tukey | 0.781299 | 0.998293 | Hedges’ g = −0.223 | −2.073 to 1.423 |
|  | TLR4/β-actin | Pp-14d vs Pp-TCBZ | Tukey | 0.395483 | 0.895070 | Hedges’ g = 0.418 | −1.820 to 4.829 |
|  | TLR4/β-actin | Pp-28d vs Pp-42d | Tukey | 0.112577 | 0.454474 | Hedges’ g = 1.557 | 0.941 to 9.783 |
|  | TLR4/β-actin | Pp-28d vs Pp-TCBZ | Tukey | 0.015497 | 0.089685 | Hedges’ g = 1.434 | 0.944 to 17.453 |
|  | TLR4/β-actin | Pp-42d vs Pp-TCBZ | Tukey | 0.267961 | 0.765905 | Hedges’ g = 0.515 | −1.049 to 3.380 |
| Figure 3C | p-p65/p65 | Ctrl vs Pp-14d | Games–Howell | 0.596792 | 0.958336 | Hedges’ g = 0.405 | −5.314 to 14.700 |
|  | p-p65/p65 | Ctrl vs Pp-28d | Games–Howell | 0.064191 | 0.236931 | Hedges’ g = −1.727 | −7.171 to −0.740 |
|  | p-p65/p65 | Ctrl vs Pp-42d | Games–Howell | 0.102987 | 0.292993 | Hedges’ g = −1.833 | −23.294 to −1.116 |
|  | p-p65/p65 | Ctrl vs Pp-TCBZ | Games–Howell | 0.930791 | 0.999960 | Hedges’ g = −0.064 | −6.803 to 7.266 |
|  | p-p65/p65 | Pp-14d vs Pp-28d | Games–Howell | 0.029135 | 0.089680 | Hedges’ g = −3.546 | −33.069 to −2.639 |
| Figure 3C | p-p65/p65 | Pp-14d vs Pp-42d | Games–Howell | 4.62 × 10⁻⁵ | 0.000218 | Hedges’ g = −13.227 | −137.206 to −11.510 |
|  | p-p65/p65 | Pp-14d vs Pp-TCBZ | Games–Howell | 0.024730 | 0.099203 | Hedges’ g = −2.455 | −13.516 to −1.794 |
|  | p-p65/p65 | Pp-28d vs Pp-42d | Games–Howell | 0.628569 | 0.970506 | Hedges’ g = 0.366 | −4.153 to 6.881 |
|  | p-p65/p65 | Pp-28d vs Pp-TCBZ | Games–Howell | 0.040963 | 0.127855 | Hedges’ g = 2.843 | 1.994 to 22.806 |
|  | p-p65/p65 | Pp-42d vs Pp-TCBZ | Games–Howell | 0.000231 | 0.001080 | Hedges’ g = 8.648 | 7.625 to 30.557 |
| Figure 3D | p-IκB/β-actin | Ctrl vs Pp-14d | Dunn | 0.082837 | 0.828374 | Cliff’s δ = −1.000 | −1.000 to −1.000 |
|  | p-IκB/β-actin | Ctrl vs Pp-28d | Dunn | 0.035764 | 0.357638 | Cliff’s δ = −1.000 | −1.000 to −1.000 |
|  | p-IκB/β-actin | Ctrl vs Pp-42d | Dunn | 0.013711 | 0.137108 | Cliff’s δ = −1.000 | −1.000 to −1.000 |
|  | p-IκB/β-actin | Ctrl vs Pp-TCBZ | Dunn | 0.927264 | 1.000000 | Cliff’s δ = −0.111 | −1.000 to 1.000 |
|  | p-IκB/β-actin | Pp-14d vs Pp-28d | Dunn | 0.715001 | 1.000000 | Cliff’s δ = −0.333 | −1.000 to 0.778 |
|  | p-IκB/β-actin | Pp-14d vs Pp-42d | Dunn | 0.465209 | 1.000000 | Cliff’s δ = −0.556 | −1.000 to 0.333 |
|  | p-IκB/β-actin | Pp-14d vs Pp-TCBZ | Dunn | 0.100348 | 1.000000 | Cliff’s δ = 1.000 | 1.000 to 1.000 |
| Figure 3D | p-IκB/β-actin | Pp-28d vs Pp-42d | Dunn | 0.715001 | 1.000000 | Cliff’s δ = −0.333 | −1.000 to 1.000 |
|  | p-IκB/β-actin | Pp-28d vs Pp-TCBZ | Dunn | 0.044610 | 0.446097 | Cliff’s δ = 1.000 | 1.000 to 1.000 |
|  | p-IκB/β-actin | Pp-42d vs Pp-TCBZ | Dunn | 0.017622 | 0.176221 | Cliff’s δ = 1.000 | 1.000 to 1.000 |

**Note:** Pairwise comparisons were performed only after a significant omnibus test. Tukey, Games–Howell, or Dunn adjustments were applied across the ten pairwise comparisons within each outcome, as appropriate. Hedges’ *g* is reported for parametric comparisons and Cliff’s δ for nonparametric comparisons. Effect-size confidence intervals are unadjusted 95% percentile bootstrap intervals based on 10,000 within-group resamples. Effect-size signs represent the first-listed group minus the second-listed group. For qRT-PCR outcomes, pairwise statistics and effect sizes were calculated using ΔCt values. Alpha-diversity pairwise comparisons are shown in Fig. 4A and reported in the Results.

**Table H. Sequencing depth and species-level diversity quality metrics for the 30 lung tissue samples included in the microbiome analysis**

| **Group** | **Sample ID** | **Denoised sequence count** | **Observed taxa (ASV)** | **Reconstructed sequence count** | **Good’s coverage** |
| --- | --- | --- | --- | --- | --- |
| Ctrl | D0_3 | 211,557 | 210 | 28,280.88 | 1 |
| Ctrl | D0_4 | 247,716 | 152 | 16,724.82 | 1 |
| Ctrl | D0_7 | 208,387 | 185 | 22,342.24 | 1 |
| Ctrl | D0_8 | 196,934 | 239 | 24,591.29 | 1 |
| Ctrl | D0_9 | 207,269 | 252 | 34,948.95 | 1 |
| Ctrl | D0_10 | 186,779 | 261 | 32,256.61 | 1 |
| Pp-14d | D14_1 | 186,440 | 107 | 8,085.36 | 1 |
| Pp-14d | D14_2 | 146,000 | 58 | 2,663.82 | 1 |
| Pp-14d | D14_3 | 249,161 | 38 | 6,400.46 | 1 |
| Pp-14d | D14_4 | 202,948 | 57 | 3,855.92 | 1 |
| Pp-14d | D14_5 | 142,643 | 245 | 24,829.77 | 1 |
| Pp-14d | D14_7 | 136,731 | 162 | 10,143.20 | 1 |
| Pp-28d | D28_1 | 174,265 | 12 | 34,995.40 | 1 |
| Pp-28d | D28_2 | 222,876 | 14 | 114,570.93 | 1 |
| Pp-28d | D28_3 | 162,307 | 90 | 37,688.50 | 1 |
| Pp-28d | D28_5 | 154,037 | 42 | 11,342.46 | 1 |
| Pp-28d | D28_6 | 177,667 | 27 | 4,355.20 | 1 |
| Pp-28d | D28_7 | 149,396 | 51 | 4,750.06 | 1 |
| Pp-42d | D42_1 | 150,437 | 65 | 23,252.58 | 1 |
| Pp-42d | D42_2 | 149,121 | 32 | 1,863.54 | 1 |
| Pp-42d | D42_3 | 272,509 | 60 | 5,327.71 | 1 |
| Pp-42d | D42_6 | 124,225 | 23 | 6,448.54 | 1 |
| Pp-42d | D42_7 | 144,981 | 112 | 5,522.82 | 1 |
| Pp-42d | D42_8 | 175,837 | 71 | 3,207.21 | 1 |
| Pp-TCBZ | D60_3 | 151,270 | 95 | 6,013.66 | 1 |
| Pp-TCBZ | D60_4 | 154,144 | 210 | 16,217.98 | 1 |
| Pp-TCBZ | D60_6 | 213,153 | 32 | 4,497.36 | 1 |
| Pp-TCBZ | D60_7 | 179,929 | 140 | 11,316.10 | 1 |
| Pp-TCBZ | D60_8 | 213,046 | 131 | 17,842.99 | 1 |
| Pp-TCBZ | D60_9 | 210,125 | 85 | 5,606.70 | 1 |

**Note:** Denoised sequence count denotes the total number of sequences retained after denoising across the five amplified 16S rRNA regions. Observed taxa correspond to the species-level Sobs index. Reconstructed sequence count denotes the count generated after reconstruction of the five-region data; fractional values may occur because of the distribution of regional sequences during reconstruction. Reconstructed sequence counts are rounded to two decimal places. Good’s coverage values are reported as exported by the Majorbio Cloud platform.

**Fig A.** Representative H&E-stained lung fields illustrating histopathological features corresponding to the item-level score categories of 0, 1 and 2. These categories apply to individual histopathological components and do not represent the final weighted ATS lung injury score. Scale bars = 200 μm.

Item-level score category 0

Item-level score category 1

Item-level score category 2

**Fig B.** Rarefaction curves for species-level alpha-diversity indices in lung tissue samples. Rarefaction curves are shown for the Sobs and Shannon indices. Each curve represents one lung tissue sample, and colors indicate the Ctrl, Pp-14d, Pp-28d, Pp-42d, and Pp-TCBZ groups (*n* = 6 per group). The tendency of the curves to approach plateaus suggests that the available sequencing depth captured the major species-level microbial diversity detected in the analyzed samples.
